# Large-area waterproof and durable perovskite luminescent textiles

**DOI:** 10.1038/s41467-023-35830-8

**Published:** 2023-01-16

**Authors:** Tian Tian, Meifang Yang, Yuxuan Fang, Shuo Zhang, Yuxin Chen, Lianzhou Wang, Wu-Qiang Wu

**Affiliations:** 1 MOE Key Laboratory of Bioinorganic and Synthetic Chemistry, Lehn Institute of Functional Materials, School of Chemistry, Sun Yat-sen University, Guangzhou, 510006 P. R. China; 2 Instrumental Analysis and Research Center, Sun Yat-sen University, Guangzhou, 510275 P. R. China; 3 Nanomaterials Centre, School of Chemical Engineering and Australian Institute for Bioengineering and Nanotechnology, The University of Queensland, Brisbane, QLD 4072 Australia

**Keywords:** Nanoscience and technology, Energy science and technology

## Abstract

Lead halide perovskites show great potential to be used in wearable optoelectronics. However, obstacles for real applications lie in their instability under light, moisture and temperature stress, noxious lead ions leakage and difficulties in fabricating uniform luminescent textiles at large scale and high production rates. Overcoming these obstacles, we report simple, high-throughput electrospinning of large-area (> 375 cm^2^) flexible perovskite luminescent textiles woven by ultra-stable polymer@perovskite@cyclodextrin@silane composite fibers. These textiles exhibit bright and narrow-band photoluminescence (a photoluminescence quantum yield of 49.7%, full-width at half-maximum <17 nm) and the time to reach 50% photoluminescence of 14,193 h under ambient conditions, showcasing good stability against water immersion (> 3300 h), ultraviolet irradiation, high temperatures (up to 250 °C) and pressure surge (up to 30 MPa). The waterproof PLTs withstood fierce water scouring without any detectable leaching of lead ions. These low-cost and scalable woven PLTs enable breakthrough application in marine rescue.

## Abstract (web summary)

Instability of perovskites under light, moisture and temperature stress hinders their potential in real applications. Here Tian et al. demonstrate the high-throughput fabrication of large-area, flexible, color-tunable, waterproof and durable wearable luminescent textiles suitable for marine rescue.

## Introduction

Lead halide perovskites (LHPs) form a new generation of luminescent materials owing to their fascinating optoelectronic properties such as tunable bandgaps, high color purity and high photoluminescence quantum yield (PLQY)^1–13^. These properties enable a myriad of optoelectronic applications, such as lighting and displays, making a huge impact and challenging conventional light-emitting technologies and devices^10–13^. Despite recent advances in optoelectronic performances, the inherent imperfections and instability have been big obstacles to the practical application of LHPs devices. LHPs must overcome the decomposition upon exposure to light, heat and moisture. Encapsulating LHPs within polymers, metal oxides, nanosilica, porous alumina membranes, or making LHP-glass nanocomposites have been variously developed to stabilize the LHPs and impart advanced photonic functionalities^14–24^. However, these approaches entail several complicated synthetic (e.g. tailoring of chemical composition, bandgap and ligand anchoring of LHPs)^25–27^ and processing (e.g. supporting mesoporous template preparation, sol-gel preparation, coating, sintering or laser lithography) steps^28–34^. Due to the fast crystallization of LHPs, it is challenging to produce uniform perovskite thin films on a large scale by the common mass-fabrication techniques. In recent years, not only being integrated on small-area rigid panels, the state-of-the-art LHP-based light-emitting thin films or devices are also assembled into large-area, flexible and/or wearable electronics^35–40^. In particular, the electrospinning technique has been demonstrated as one of the most ideal ways to fabricate perovskite luminescent fibrous membranes^35–38^. Unfortunately, the current methodologies were limited to stabilizing the LHPs within a chosen polymer matrix featuring less functional groups, which then exhibited poor mechanical stability caused by weak interfacial interactions. In this case, the perovskite is only partially protected and its surface defects are not well-passivated, thus showing inferior PLQY and moderate stability against water invasion and UV irradiation. Nonetheless, through rational interfacial engineering by purposely designed chemical interactions to stabilize the LHP and mitigate the leaching of toxic heavy metal ions, we envision that perovskite luminescent fibers can be woven into textiles for smart wearable optoelectronics.

We present a simple, single-nozzle electrospinning method that can directly and continuously fabricate PLTs on a large-scale with high production rates of ~1500 cm^2^ h^−1^. Significantly distinguished to the previous works regarding polymer-encapsulated perovskite composites^35–38^, a new class of composited fibers with perovskites robustly encapsulated in polymers, cyclodextrin supermolecules and fluorinated hydrophobic agents was designed. Similar to other reports, the polymer acts as a fibrous matrix for loading perovskites and ensures mechanical flexibility. Beyond that, we innovatively introduced cyclodextrin supermolecules, which feature multidentate hydroxyl groups along the walls of internal and external cavities, and can strongly interact with perovskites to form a stable host-guest complex that simultaneously passivates the crystallographic imperfections in the perovskites. In the meantime, the super-hydrophobic fluorine-containing silane was incorporated, which forms strong hydrogen bonds with the cyclodextrin molecules, further strengthening the stabilization effect and providing a robust physical barrier at the outer surface against moisture invasion. The woven luminescent textiles showed great flexibility, stability and prominent optical properties (i.e. high PLQY, high color purity and uniform emission), and hence their potential application in a wide variety of flexible, wearable optoelectronics were demonstrated. We show that the combination of strategies of host-guest inclusion, multidentate interaction and multiple passivation by cyclodextrin molecules, as well as in-situ encapsulation and outer-layer physical barrier protection by fluorine-containing silanes enable the PLTs to be very stable against long-term water immersion (>3300 h, regardless of pH), high power ultraviolet light irradiation (UV; wavelength of 365 nm, a power density of 120 mW cm^−2^), high temperatures up to 250 °C, or high pressures up to 30 MPa. Up till now, none of the currently reported works regarding polymer-encapsulated perovskite composites have addressed the toxic Pb leakage issue, which would limit their practical application of perovskite-based textiles. Encouragingly, our demonstrated new composited structure ideally addressed the Pb leakage issue, with only ~3.94 ppt of Pb^2+^ ions leaching out even after dynamic water scouring of PLTs for >3300 h, making the PLTs environmentally viable and safe. To the best of our knowledge, our demonstrated PLTs represented one of the most promising overall performances in terms of luminescent properties (PLQYs, narrow FWHM), stability against water/heat/pressure, scalability, cost advantage and safety, etc.

## Results

### Composites synthesis and chemical interaction analysis

CsPbBr_3_@hydroxypropyl-β-cyclodextrin (HPβCD) composites were first synthesized mechanochemically by grinding. The HPβCD molecule has distinct structural features in terms of its hydrophobic inner cavity (ca. 0.78 nm in diameter, Supplementary Fig. 1)^41,42^, which can be used as host matrices for forming host-guest composites, such as CsBr@HPβCD and PbBr_2_@HPβCD. During continuous grinding, we expected that CsBr@HPβCD and PbBr_2_@HPβCD composites could completely react to form CsPbBr_3_@HPβCD composites. In this case, the HPβCD would self-aggregate and copolymerize to form a network of clusters with mesoscale and/or macroscale cavities, which can spatially limit the crystal growth and confine the particle sizes of the evolved CsPbBr_3_ nanocrystals. The HPβCD clusters were spontaneously extruded out to the surface of the CsPbBr_3_ nanocrystal to form an ingenious stable host-guest complex, which simultaneously stabilized and passivated the surface states of CsPbBr_3_. Grinding CsBr, PbBr_2_ and HPβCD blended powder significantly improved the color purity and enhanced the luminescent brightness by more than four-fold, relative to a ground CsBr and PbBr_2_ blended powder (Supplementary Fig. 2). X-ray photoelectron spectroscopy (XPS) and Fourier Transform Infrared spectroscopy (FTIR) characterizations were conducted to identify the chemical interaction between HPβCD and CsPbBr_3_ and verify the specific passivation function of HPβCD on CsPbBr_3_. The XPS results showed the Pb 4 *f* signal peak shifted to higher binding energy upon HPβCD incorporation (Supplementary Fig. 3), suggesting the change of electronic cloud density surrounding Pb^2+^ owing to the strong coordination bonding between electron-donating -OH group in HPβCD and under-coordinated Pb^2+^ ions in perovskites. Such a chemical interaction between Lewis base-typed additives and perovskites has been widely reported^43^. In addition, we expected that many positively charged hydrogen ions of HPβCD could also strongly interact with negatively charged halide ions in perovskites via electrostatic interaction and/or hydrogen bonding^44^. To further verify the bonding status of H element in HPβCD, the two-dimensional (2D) ^1^H − ^1^H nuclear Overhauser effect spectroscopy (NOESY) cross-peak intensity was measured, which can clearly distinguish between free and bound HPβCD molecules. Free HPβCD molecules behave like small molecules and develop slightly negative or even no nuclear Overhauser effect (NOE) due to a small negative or zero cross-relaxation rate (Supplementary Fig. 4)^45^. However, if HPβCD cluster interacted and was bound to the CsPbBr_3_ surface, it will develop large and positive NOEs due to a large and positive cross-relaxation rate^46,47^. The ^1^HNMR of HPβCD was estimated by ChemDraw 19.0 software (Supplementary Fig. 5a). NOESY spectra of CsPbBr_3_@HPβCD samples showed many positive cross peaks at ～3.34 ppm (Supplementary Fig. 5b), which indicated a large portion of intramolecular hydrogen bonds forming between HPβCD clusters with multidentate functional groups and CsPbBr_3_ perovskites. Overall, the NMR and XPS results synergistically verified the strong interaction between HPβCD molecules/clusters and CsPbBr_3_, which is beneficial to effectively passivate the undercoordinated metal cations and stabilize the halide anions (reduce the halide vacancies and/or mitigate their migration) in CsPbBr_3_.

### Fabrication of large-area PLTs and morphological characterization

**Fig. 1:**
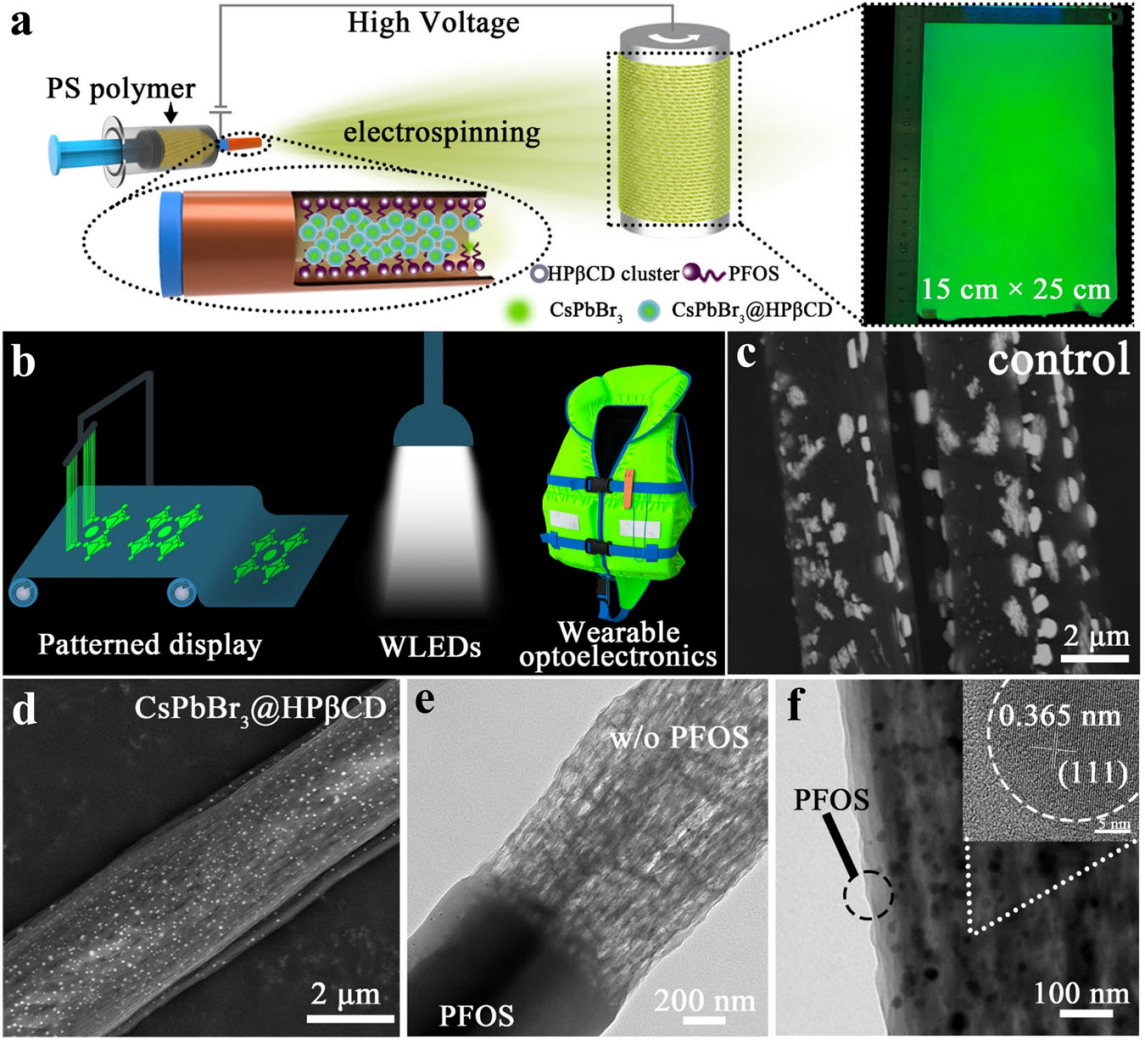
Schematic illustration of the fabrication process of PLTs and morphological characterization. **a** Schematic illustration of the fabrication process of PLTs and the as-prepared fibrous membrane under UV light irradiation. **b** Application of CsPbBr_3_@HPβCD@PFOS composites in patterned display, white light-emitting diodes (WLEDs) and wearable optoelectronics. SEM (YAG back-scattered electron detector) images of the (**c**) control fiber and (**d**) CsPbBr_3_@HPβCD fiber. **e** TEM image showing the CsPbBr_3_@HPβCD fiber without or with PFOS coating as indicated. Note: the top half of the CsPbBr_3_@HPβCD@PFOS fiber was selectively etched by n-hexane to expose the inner CsPbBr_3_@HPβCD. **f** Close-up TEM image of the CsPbBr_3_@HPβCD@PFOS fiber. Inset: HRTEM image of the CsPbBr_3_ crystal.

The aggregation of CsPbBr_3_ nanocrystals (Supplementary Fig. 6a, b) were effectively suppressed in the CsPbBr_3_@HPβCD composites, in which the CsPbBr_3_ nanocrystals were uniformly encapsulated and spatially isolated by HPβCD clusters (Supplementary Fig. 6c). We identified CsPbBr_3_ nanocrystals as the major luminescent source within the composite fragment, and there is a portion of light scattering to the surrounding HPβCD clusters (Supplementary Fig. 6d). Note, it is the most ideal case that the CsPbBr_3_ crystal was fully encapsulated in HPβCD cluster. In the real case, there is still some chance that a CsPbBr_3_ crystal is half-coated in an HPβCD cluster, in which the sizes of perovskite crystals and/or the cavities of self-formed HPβCD clusters should be carefully controlled. After grinding, we mixed CsPbBr_3_@HPβCD powder, polystyrene (PS) and perfluorooctyltriethoxysilane (PFOS) at designed weight ratios in a binary solvent of N,N′-dimethylformamide (DMF) and dimethyl sulfoxide (DMSO) to prepare the electrospinning ink, which was ready to be used for large-area PLTs fabrication via single-nozzle electrospinning (Fig. 1a and Supplementary Fig. 7, see details in the “Methods” section of Supplementary Materials). Note, rather than prior mechanochemical grinding, the electrospinning ink can also be prepared by directly mixing CsBr, PbBr_2_, HPβCD, PS and PFOS ingredients together and stirring for 6 h, which then can be directly used to fabricate the large-area PTLs via electrospinning (Supplementary Fig. 8). And the mechanochemical grinding step is intentionally designed to obtain the CsPbBr_3_@HPβCCD composited powders and elucidate the beneficial roles of HPβCD inclusion and passivation on improving the luminescent properties of CsPbBr_3_. A 25 cm × 15 cm PLT was produced within 30 min, which had uniform green light emission over the whole display area (Fig. 1a and Supplementary Fig. 9), demonstrating a great potential to be used in large-area patterned displays, flat-panel lighting and other wearable optoelectronics (Fig. 1b). Note, the previously reported electrospinning techniques still require to use 2 or 3 nozzles to load each individual ingredient, such as metal halides, inorganic/organic halide salts and polymers, which is complicated and hard to control the electrospinning parameters and reaction process^35–38^. In our case, we simply relied on using single-nozzle containing all the ingredients including perovskites, polymers, cyclodextrins and silanes to achieve high-throughput fabrication of high-quality PLTs, which represents a technological advance, especially in terms of reducing fabrication cost and being more compatible to the industrial-scale mass production. Fluorescence confocal microscopy confirmed the fibrous morphology and verified the fluorescence characteristic in the targeted PLT. A consistent, bright fluorescence was detected from individual fibers, inferring the uniform dispersion of effective luminescent perovskite components (Supplementary Fig. 10). An electrospinning process lasting 3 min yielded a similar-sized textile with observable light emission (Supplementary Fig. 11 and Supplementary Movie #1), suggesting the possibility of realizing high-throughput mass production. Indeed, electrospinning is a process of densification, with increased numbers of fibers well-interconnected and woven into a high density but lightweight fibrous network (i.e. ~10–100 mg for a ~375 cm^2^ textile). The electros pinned PLTs also have superior advantages in terms of reduced material cost and simplified fabrication process. To weave a 2.8 m textile (280 cm × 15 cm, Supplementary Fig. 12), only 0.464 g CsPbBr_3_ powder, 0.104 g of HPβCD, 1.2 ml of PFOS, 1.22 g of PS and 8 ml of mixed DMF-DMSO solvent were needed. In addition, the PLTs fabrication does not require tedious procedures, such as ligand exchange, solvent washing and high-temperature processing. Overall, the cost of the luminous textile was calculated to be as low as ~0.05 cents cm^−2^, demonstrating the prospect of low-cost mass production (Supplementary Table 1).

To elucidate the specific role of the PS polymer, cyclodextrin and silane, we investigated the morphologies of different PLT fibrous membranes. During the electrospinning process, the PS polymer served as a fibrous scaffold holding the CsPbBr_3_ nanocrystals within the matrix. However, numerous irregular-shaped CsPbBr_3_ crystals were randomly distributed and aggregated on the surface of the fibrous composites (Fig. 1c, Supplementary Fig. 13a, b). In contrast, the fibrous film made from the CsPbBr_3_@HPβCD composites showed more uniform dispersion of perovskite nanocrystals with smaller sizes inside and/or at the surface of the polymer matrix (Fig. 1d), which further strengthened the quantum confinement effect and might induce blue-shift of absorption band edge^14,48^. The CsPbBr_3_@HPβCD fibrous films are composed of uniform fibers with hierarchical structures and diameters of several micrometers; each microfiber consists of tiny nanowires with diameters of tens of nanometers (Supplementary Fig. 13c, d). However, the exposure of CsPbBr_3_ nanocrystals on the membrane surface is unfavorable for ensuring the good water resistance of the CsPbBr_3_@HPβCD fibrous film, thus limiting its practical use and shortening its lifespan. To address this issue, PFOS was incorporated as a separated nanophase ingredient into CsPbBr_3_@HPβCD composites during the electrospinning process. On the one hand, PFOS could be spontaneously expelled and fully cover the outer surface of the fibers, so that exposed CsPbBr_3_ nanocrystals were not observed (Fig. 1e, Supplementary Fig. 13e, f). On the other hand, the hydrophobic PFOS molecules with many fluorine (F) atoms can form strong hydrogen bonds with the numerous –OH groups in HPβCD, thus constructing a seamless interfacial contact with the CsPbBr_3_@HPβCD composite fibers, which, crucially serves as a robust protection layer (~ 50 nm in thickness, Fig. 1f) to enhance the waterproofness of the nanofiber composited film. The CsPbBr_3_ nanocrystals with the average particle size of 14.24 ± 6.02 nm are uniformly dispersed in the nanofibers (Fig. 1f and Supplementary Fig. 14). The crystalline and amorphous regions of CsPbBr_3_@HPβCD@PFOS composite fibers were identified by HRTEM (inset in Fig. 1f). The crystalline regions exhibit distinct and clear lattice fringes (i.e. a distance of 0.356 nm), corresponding to the (111) crystal planes of highly crystalline CsPbBr_3_, which reside within the amorphous cyclodextrin and silane surroundings. Note, the CsPbBr_3_@HPβCD nanocrystals can be obtained by dissolving the CsPbBr_3_@HPβCD fibers in chlorobenzene (CB) solution and then undergoing ultrasonic dispersion for 20 min. The HRTEM image clearly showed the encapsulation of crystalline CsPbBr_3_ nanocrystals in amorphous HPβCD matrix (Supplementary Fig. 15). Besides, the elemental mapping of these three kinds of fibers was conducted by using energy dispersive spectroscopy mapping function in SEM or HRTEM (Supplementary Fig. 16-18), and the corresponding element distribution data was summarized in Supplementary Table 2–4. The results showed that the HPβCD and PFOS play important roles in promoting the uniform distribution of CsPbBr_3_ nanocrystals within the polymer skeleton and maintaining the stoichiometric ratio of Cs, Pb and Br (close to 1:1:3).

### Chemical interaction mechanism and water-proof property

**Fig. 2:**
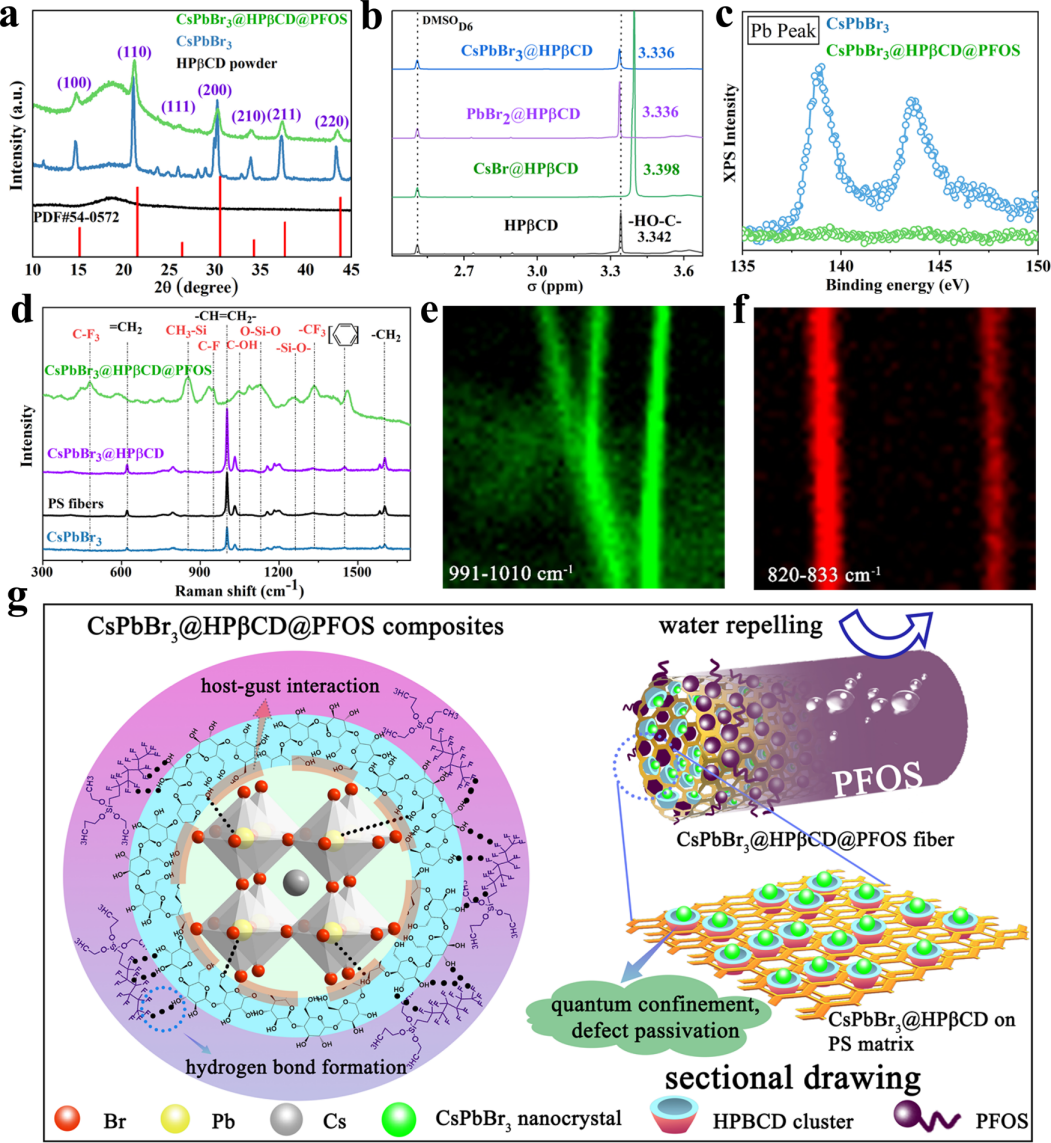
Chemical interaction, bonding formation and multifaceted functions of PLTs. **a** XRD patterns of HPβCD power, CsPbBr_3_ fibers and CsPbBr_3_@HPβCD@PFOS fibers. **b** The ^1^HNMR spectra of HPβCD, CsBr@ HPβCD, PbBr_2_@HPβCD and CsPbBr_3_@HPβCD. **c** XPS spectra of the Pb signal of the CsPbBr_3_ fibers and CsPbBr_3_@HPβCD@PFOS fibers. **d** Raman spectrum of PS, CsPbBr_3_, CsPbBr_3_@HPβCD and CsPbBr_3_@HPβCD@PFOS fibers. Raman confocal fluorescence mapping of(**e**) CsPbBr_3_ fibers and (**f**) CsPbBr_3_@HPβCD@PFOS fibers. **g** Schematic illustration of the chemical interaction of CsPbBr_3_@HPβCD@PFOS composites and their multifaceted functions.

HPβCD interaction and PFOS encapsulation did not alter the chemical identity of CsPbBr_3_, as revealed by X-ray diffraction (XRD) patterns (Fig. 2a) that showed characteristic (100), (110), (111), (200), (210), (211) and (220) planes of cubic CsPbBr_3_ for all fibrous films (PDF 54-0752)^49^. Specifically, HPβCD incorporation could facilitate the reaction between the PbBr_2_ and CsBr raw materials, diminishing any excess metal halide residues (Supplementary Fig. 19). The slight broadening of diffraction peaks in the CsPbBr_3_@HPβCD@PFOS fibers can be ascribed to the amorphous characteristic of the HPβCD and PFOS components. ^1^H nuclear magnetic resonance (^1^H-NMR) spectroscopy provided insights into possible chemical interactions between HPβCD and different species, CsBr, PbBr_2_ or CsPbBr_3_. The chemical identity of H-3 in the HPβCD molecule is 3.341 ppm, which represents the H atoms located at the hydrophobic inner cavity of HPβCD. After grinding and interaction with CsBr, PbBr_2_ or CsPbBr_3_ species, the ^1^H spectra all shifted, but to different extents. A greater chemical shift was observed for CsBr@HPβCD sample (Fig. 2b), owing to the shorter molecular length of CsBr (~0.35 nm) relative to PbBr_2_ (~0.52 nm) (Supplementary Fig. 20), making it much easier to be captured within the HPβCD inner cavity. We also performed XPS to reveal the additional protection efficacy that PFOS has on the CsPbBr_3_@HPβCD fibrous film. The control CsPbBr_3_ nanofibers exhibited notable signals for the three main elements, Cs, Pb and Br, suggesting that CsPbBr_3_ was not fully encapsulated by the polymer matrices (Fig. 2c and Supplementary Fig. 21), which is consistent with the SEM observation (Fig. 1c). In contrast, these elemental signals were all diminished in the CsPbBr_3_@HPβCD@PFOS fibrous films, hinting at the full coverage and omnibearing protection of the PFOS layer. Similar characteristic signals have been observed in the Raman spectra of PS, CsPbBr_3_ and CsPbBr_3_@HPβCD fibrous films, which mostly stem from the Raman shift signals of PS polymer matrices. Again, these PS-related signals disappeared in our CsPbBr_3_@HPβCD@PFOS sample (Fig. 2d). The PFOS molecule is embedded with highly polarized C-F bonds and is amphiphilic by nature, meaning it is easy to form strong H-F bonds with numerous -OH groups in HPβCD and expose the superhydrophobic moieties. The hydrogen bonding formation between the PFOS and HPβCD was characterized and analyzed by FTIR (Supplementary Fig. 22) and ^1^HNMR (Supplementary Fig. 23 and Supplementary Table 5). Specifically, the FTIR result showed the broadened signals of -OH, C-F and CF_2_/CF_3_ groups in the HPβCD@PFOS sample, relative to pristine HPβCD or PFOS sample, which implied the alteration of bonding environment owing to the formation of hydrogen bonds. The ^1^HNMR spectroscopy further verified the chemical interaction between HPβCD and PFOS. The positions of protons in HPβCD are illustrated in Supplementary Fig. 23, in which the H_2_, H_4_ and H_6_ protons located at the outer surface of HPβCD, and the H_3_ and H_5_ located at the inner cavity of HPβCD. Upon interacting with PFOS, the resonances of H_2_, H_3_, H_4_, H_5_ and H_6_ protons in HPβCD molecule all shifted towards high-field positions owing to the formation of hydrogen bonds (Supplementary Table 5). Typically, the chemical shift of H_2_, H_4_ and H_6_ protons is comparatively larger than that of the H_3_ and H_5_ protons, indicating that the hydrogen bonds were mainly formed between the F atoms of PFOS molecules and the -OH groups of HPβCD outer surface. This result is in good consistency with our demonstrated chemical interaction modes in Fig. 2g (left). With the PFOS protection, a robust water-repelling barrier was established on the surface of PLTs, as is evidenced by the >130° water contact angle (Supplementary Figs. 24 and 25). An optimal content of PFOS (i.e. 150 μL in 1 mL electrospinning ink) ensured the best luminescent property and hydrophobic performance (Supplementary Fig. 26). Raman confocal fluorescence mapping revealed stretching of the -CH = CH_2_- (991–1010 cm^−1^) of PS in the CsPbBr_3_ fiber (Fig. 2e), while the CsPbBr_3_@HPβCD@PFOS fiber showed only stretching and bending of the -C-F (820–833 cm^−1^) of PFOS, and without detectable PS-related signals (Fig. 2f)^50^. The host-guest interaction between the CsPbBr_3_ nanocrystal and HPβCD clusters, hydrogen bonding formation between HPβCD and PFOS, as well as the multifaceted functions including quantum confinement, multiple defect passivation and water-repellent effect are schematically illustrated in Fig. 2g.

### Optical property

**Fig. 3:**
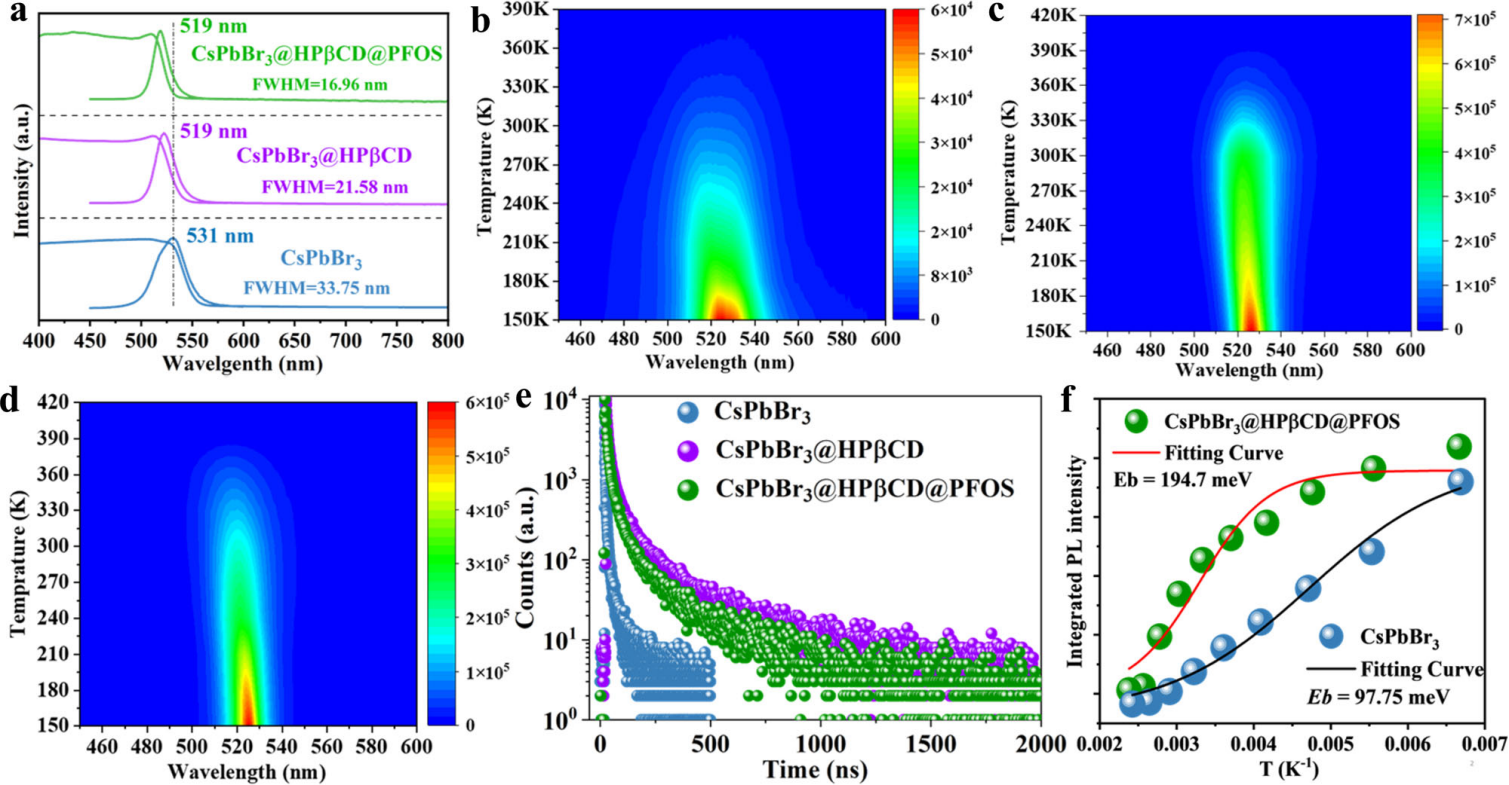
The characterization of optical property, temperature-dependent photoluminescence and exciton binding energy of different PLTs. **a** UV-visible absorption and PL spectra of CsPbBr_3_, CsPbBr_3_@HPβCD and CsPbBr_3_@HPβCD@PFOS fibers. Temperature-dependent PL mapping of (**b**) CsPbBr_3_, (**c**) CsPbBr_3_@HPβCDand (**d**) CsPbBr_3_@HPβCD@PFOS fibers. **e** Time-resolved PL decay of CsPbBr_3_, CsPbBr_3_@HPβCD and CsPbBr_3_@HPβCD@PFOS fibers measured at 300 K. **f** Arrhenius plot of integrated PL intensity as a function of the reciprocal temperature (1/T) of CsPbBr_3_ and CsPbBr_3_@HPβCD@PFOS fibers.

Incorporation of HPβCD and PFOS gradually bleached the textiles from yellow to white, and progressively enhanced the luminescent brightness (Supplementary Fig. 27), which can be attributed to effective molecular interaction and encapsulation. Specifically, all of the CsPbBr_3_-based fibrous films showed green PL emission, with the strongest PL intensity obtained with CsPbBr_3_@HPβCD@PFOS membranes upon excitation by a 420 nm laser (Supplementary Fig. 28). HPβCD and PFOS modification blue-shifted the PL maxima, which we attribute to the formation of smaller CsPbBr_3_ nanocrystals16^51^. The modification also led to reduced PL FWHM (Fig. 3a), which was concomitant with an observed enhanced homogeneity for the CsPbBr_3_ nanocrystals, namely, uniform in size and well-dispersed in the fibrous matrix (Fig. 1f). The overlap of the absorption band edge and PL emission peak is effectively mitigated by HPβCD and PFOS incorporation, which is desirable to suppress self-absorption and improve luminescence. As a result, the CsPbBr_3_@HPβCD@PFOS fibers showed a bright and narrow-band green light emission with high color purity, regardless of excitation wavelength variation from 390 to 440 nm (Supplementary Fig. 29). The enhanced luminescent property and mechanism can be understood through monitoring the carrier dynamics by temperature-dependent PL intensities and lifetimes characterization. Compared with its CsPbBr_3_ counterpart, the CsPbBr_3_@HPβCD@PFOS fibrous film exhibited a more than fourfold enhancement of radiative recombination rate (*K*_rad_) and a 25-fold improvement of PLQY (~50%) (Supplementary Table 6). In addition, the CsPbBr_3_@HPβCD and CsPbBr_3_@HPβCD@PFOS fibrous films always exhibited stronger PL intensity, narrower FWHM and longer excited-state PL lifetimes when the temperature varied from 150 K to above 390 K (Fig. 3b–e, Supplementary Fig. 30), suggesting significantly improved thermal stability. This result was further confirmed by temperature-dependent XRD testing, which showed that the CsPbBr_3_@HPβCD@PFOS fibrous film maintained its crystal structure and composition at high temperature up to 250 °C (Supplementary Fig. 31). During the temperature-dependent PL test, the PL intensities of all the PLTs gradually decreased as the temperature increased, which is a common phenomenon for luminescent materials, especially when the thermal energy is high enough to induce excitons dissociation and facilitate electron-phonon interaction, thus causing significant PL quenching^48^. In addition, one could also notice more obvious blue-shift of PL peak when increasing the temperature from 150 to 300 K, which could be attributed to the fact that, at this low temperature zone, the thermal motion and expansion dominated, which resulted in the bandgap enlargement of semiconducting materials^52,53^ To better verify the thermal stability of CsPbBr_3_@HPβCD@PFOS fibrous film, an additional new set of temperature-dependent PL measurements was conducted by heating the sample from 290 K (near room temperature) to 410 K and then cool down to 290 K. As a result, the PL peak did not blue-shift any more (Supplementary Fig. 32a, b). More interestingly, though one could still notice the PL quenching effect upon heating, the PL intensity was able to recover and became even stronger than before when the sample was subsequently cooled down from 410 K to 290 K (Supplementary Fig. 32c, d). This result suggested our CsPbBr_3_@HPβCD@PFOS fibrous film is indeed thermally stable and the PL quenching effect caused by high temperature-induced excitons dissociation is recoverable. For the temperature-dependent PL lifetimes, they also fluctuated with temperature variation. The PL lifetimes were fitted by a triexponential decay function:

1 $$A\left(t\right)={A}_{1}{{{{{\rm{e}}}}}}{xp}\left(-\frac{t}{{\tau }_{1}}\right)+{A}_{2}{{{{{\rm{e}}}}}}{xp}\left(-\frac{t}{{\tau }_{2}}\right)+{A}_{3}{{{{{\rm{e}}}}}}{xp}\left(-\frac{t}{{\tau }_{3}}\right)$$

At temperatures above 360 K, the CsPbBr_3_@HPβCD@PFOS fibrous film showed a remarkably longer PL lifetime (i.e. 90. 48 ns) than the other two candidates (i.e. 6.32 ns for CsPbBr_3_ and 67.90 ns for CsPbBr_3_@HPβCD, Supplementary Fig. 30 and Tables 7–9), further verifying the better thermal stability of the former. This result highlighted the synergistic and beneficial roles of HPβCD inclusion and PFOS encapsulation in mitigating the excitons dissociation and electron−phonon interaction in CsPbBr_3_ perovskites under high thermal stress. The exciton binding energy (*E*_b_) was calculated from the Arrhenius plot, according to the following equation:^54^

2 $$I\left(T\right)=\frac{{I}_{0}}{1+A\,\exp \left(\frac{{E}_{b}}{{K}_{B}T}\right)}$$

Where *I*_0_ is the integrated PL intensity at 0 K, *K*_B_ is the Boltzmann constant and *A* is the Arrhenius coefficient. The CsPbBr_3_@HPβCD@PFOS composite film had a larger *E*_b_ value (194.7 meV) than that of its CsPbBr_3_ counterpart (97.75 meV) (Fig. 3f). This suggested a more effective inhibition of exciton dissociation due to the synergic effects of nanoconfinement, encapsulation and special isolation induced by both HPβCD and PFOS modification, thus facilitating the radiative recombination rate and improving the luminescent brightness and PLQYs. Other factors, such as a high surface-to-volume ratio and limited carrier diffusion length of small CsPbBr_3_ nanocrystals, suppressed ion migration from quantum dots to quantum dots, well-passivated surface trap-states through HPβCD-assisted home-guest inclusion, and reduced halide deficiency via PFOS-assisted halogen compensation^55^, could contribute together to reduce non-radiative recombination and allow the exciton to recombine via a radiative pathway, thus emitting strong narrow luminescence from isolated crystals.

### Stability evaluation

The LHPs are notorious for their intrinsic instability that can occur through light-, thermal- and chemical-induced decomposition, which makes LHPs susceptible to PL quenching^52,56,57^. We investigated the stability of PLTs in diverse environmental and operational settings, including UV irradiation, ambient storage or water immersion. The CsPbBr_3_@HPβCD@PFOS composited textile exhibited outstanding stability for exposure under 24 h continuous UV irradiation (a power density of 120 mW cm^−2^), over 5000 h storage in ambient air (that is, at ~20–30 °C and 50–80% relative humidity) and the extrapolated time to drop down to 50% PL (*T*_PL50_) is 14,193 h (Supplementary Figs. 33–35). On the one hand, this high ambient stability originates from the effective protection of the encapsulating PFOS layer that enhances the hydrophobicity of the fibrous film and prevents invasion of perovskite by ambient oxygen and water, thus effectively circumventing PL quenching caused by composition decomposition or interaction with external agents in the surrounding environment. On the other hand, the CsPbBr_3_ nanocrystals were well-immobilized and stabilized in the polymeric matrix and HPβCD cavities through host-guest inclusion and interaction, and hence the ion diffusion was effectively suppressed, accounting for the substantially extended lifespan under UV light irradiation or thermal stress.

### Applications of PLTs

**Fig. 4:**
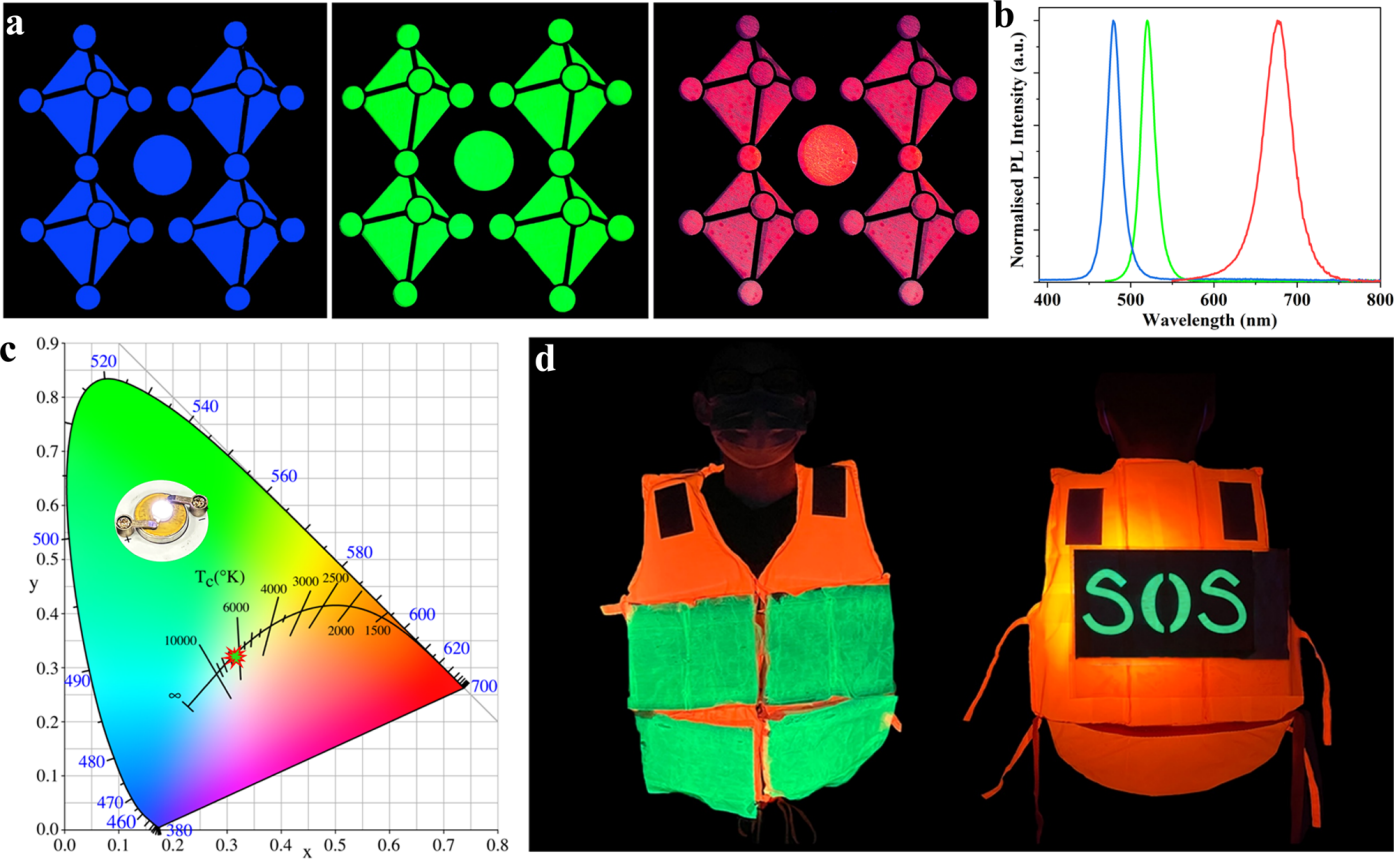
Applications of PLTs in patterned display, WLEDs and wearable luminescent clothes. **a** Patterned fluorescent displays (10 cm × 10 cm) and (**b**) their corresponding PL spectra of different colors (blue, green and red). **c** The 1931 CIE chromaticity coordinates of white LEDs fabricated with CsPbBr_3_@HPbCD@PFOS composites. (The working voltage is 5 V and the driving current is 100 mA.). **d** A demonstration of a life jacket with “SOS” patterned PLT (10 cm × 22 cm) sewed on it.

For conventional LHP-based light-emitting devices, nanocrystals with different emission wavelengths are deposited on small-size, rigid substrates, and patterning is very difficult. Electrospinning polymer@perovskite@cyclodextrin@silane composites allow for application in high-resolution patterned displays with bright luminescence and wide color gamut. To demonstrate the versatility of weaving color-tunable PLTs, all-inorganic CsPb(Cl/Br)_3_ and CsPb(Br/I)_3_-based PLTs were fabricated via a similar composite methodology and electrospinning process (see the details in the “Methods” of Supplementary Materials). Figure 4a, b shows large-area, patterned perovskite fluorescent displays (10 cm × 10 cm) of different colors (i.e. blue, green and red) that exhibiting excellent uniformity and brightness. It is worth pointing out the red perovskite composited fibrous film also showcased good light stability, which can preserve 73.2% of initial PL intensity after UV light irradiation for 12 h (Supplementary Fig. 36). Through combining commercially available BaMgAl_10_O_17_:Eublue-emitting powder, KSF (K_2_SiF_6_:Mn^4+^) red phosphor, CsPbBr_3_@HPβCD@PFOS and a UV chip, our demonstrated monolithic composites can be applied to fabricate white LEDs, showing a color temperature of 6388 K and a color-rendering index of 72.5. The Commission International L’Eclairage (1931CIE) chromaticity coordinates are (0.316, 0.318) (Fig. 4c). Simultaneously, the white LEDs can also be fabricated by stacking all PLTs that emitted blue, green and red light (see Supplementary Fig. 37), which showed a color temperature of 8079 K and a color-rendering index of 67.5. The Commission International L’Eclairage (1931CIE) chromaticity coordinates are (0.2822, 0.3421). Given that the woven PLTs are flexible, lightweight, durable, and have fascinating luminescent properties and robust mechanical strength (Supplementary Movie # 2), we envision a great potential for them to be used as wearable luminescent clothes in marine rescue or night work safety. As a proof-of-concept, we demonstrated a life jacket with a large-area (10 cm × 22 cm) “SOS” pattern and other sheets sewed on it (Fig. 4d), which could send a macroscopic SOS signal when people are encountering danger in a marine environment.

**Fig. 5:**
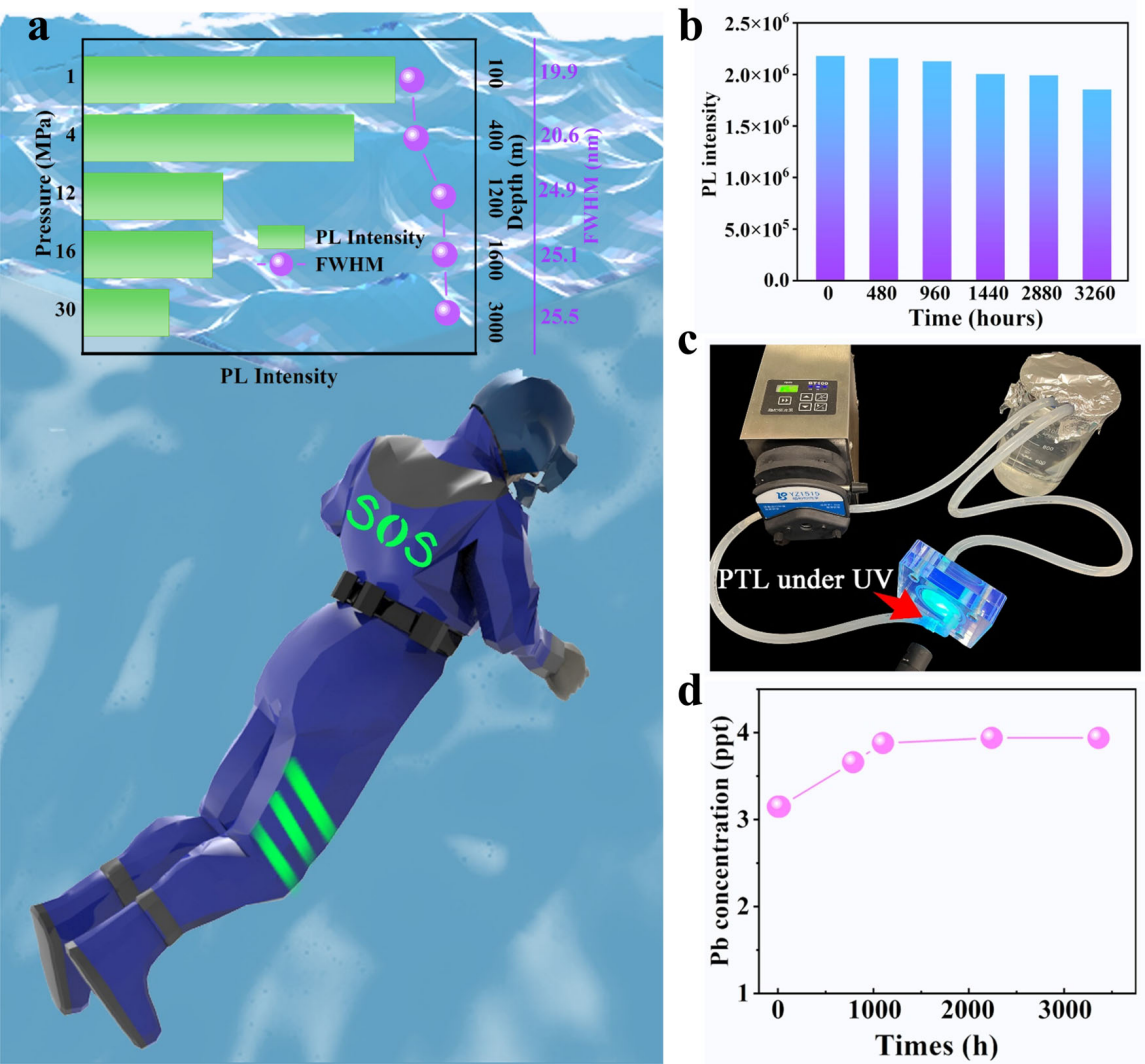
Demonstration of PLT in marine rescue. **a** The evolution of PL intensity and FWHM of CsPbBr_3_@HPbCD@PFOS-based PLTs as a function of pressure and depth, and the schematic image of an “SOS” signal patterned on the life jacket made by CsPbBr_3_@HPbCD@PFOS fiber. **b** The evolution of PL intensity of CsPbBr_3_@HPbCD@PFOS-based PLTs during 3260 h of water immersion. **c** The setup to simulate the real conditions of PLTs under dynamic seawater scour. **d** A trace of leaked Pb ions concentrations after extended scouring time.

Considering that the marine environment and real circumstances are complex and dynamic (e.g. water flow, pH values and depth), we purposely investigated the acid/base resistance of PLTs and evaluated their safety and reliability in terms of sequestrating toxic components. The CsPbBr_3_@HPβCD@PFOS textile preserved ~63–73% of PLQY when immersed in water of pH = 1.2 (PLQY = 31.4%) and pH = 12.8 (PLQY = 36.3%) for 24 h (Supplementary Figs. 38 and 39). We also evaluated the tolerance of PLTs upon high pressure extrusion (related to the seabed depth), when a rescuer dives deep into the sea. When pressure increases to 30 MPa (equivalent to 3000 meters in depth), the PLT still preserved 38% of its initial PL intensity, with a slightly enlarged FWHM (Fig. 5a). Our demonstrated CsPbBr_3_@HPβCD@PFOS-based PLT showed strong water resistance, which preserved more than 85% PL intensity even after 3260 h immersion (Fig. 5b), and the extrapolated *T*_PL50_ is ~12,189 h (Supplementary Fig. 35). A home-made apparatus was used to simulate the real conditions of PLTs under dynamic seawater scour at a fast flow rate of 570 mL min^−1^, during which the PLT could continuously emit green light upon excitation (Fig. 5c and Supplementary Movie # 3). As revealed by inductively coupled plasma-mass spectrometer (ICP-MS) measurement, a negligible Pb^2+^ concentration of 3.94 ppt (Fig. 5d) was detected after dynamic scouring for 3300 h, which is 8 orders of magnitude lower than the regulated Pb content in drinking-water (<0.01 ppm) according to the World Health Organization guidelines. This excellent capability of inhibiting Pb ions leakage can be ascribed to the in-situ encapsulation and effective chemisorption of heavy metal ions by HPβCD cluster. In addition, the polymer matrices and outer PFOS physical barrier can effectively fix Pb^2+^ ions on the resin/nanosilane composites. In this case, the Pb^2+^ was not easily leached out even when scoured by high-flow rate water, making our demonstrated PLTs environmentally viable and safe. To the best of our knowledge, our demonstrated CsPbBr_3_@HPβCD@PFOS composites-based PLTs exhibit exceptional luminescent properties and stability, which offered substantial advantages over other reported state-of-the-art analogues (Supplementary Table 10).

## Discussion

We present a new type of large-area, functional luminescent textiles fabricated by electrospinning of polymer@perovskite@cyclodextrin@silane composites. The PLTs are waterproof and durable in a variety of harsh conditions, offering substantial advantages (i.e. high PLQY, high color purity and good stability) over other LHP composites reported to date. Our demonstrated approach rationally combined the strategies of host-guest chemical interaction, inclusion-like encapsulation and physical blocking, and smartly integrated them into a single fabrication process, which remarkably advances the fabrication of perovskite materials and devices with high throughput and high scalability in a cost-effective manner. We show that such a flexible, stable, durable and safe luminescent textile can be used in a variety of optoelectronic applications, such as high-resolution patterned displays, white LEDs and wearable fluorescence products for marine rescue. This work provides a new perspective for fabricating high-performance perovskite luminescent materials with low-cost advantage and safety warranty. We anticipate that this finding will promote the large-scale production and practical applications of luminescent textiles for next-generation smart and wearable optoelectronics.

## Methods

### Materials

Cesium bromide (CsBr), Cesium chloride (CsCl), lead chloride (PbCl_2_) and Iodine Cesium (CsI_2_) were purchased from Xi’an Polymer Light Technology Corp. Lead bromide (PbBr_2_, 99.9%) and lead Iodine (PbI_2_) were purchased from Aladdin. The N,N-dimethylformamide (DMF, AR), n-hexane and dimethyl sulfoxide (DMSO) were purchased from Shanghai Yinli Science and Technology Co., Ltd. Perfluorooctyltriethoxysilane (PFOS) was purchased from Heowns Industrial Corporation. Hydroxypropyl-β-cyclodextrin (HPβCD), polystyrene (PS) and deuterated dimethylsulfoxide (DMSO–d_6_) were purchased from Shanghai Macklin Biochemical Co., Ltd. All chemicals were used as received without further purification. The commercial BaMgAl_10_O_17_:Eu blue-emitted powder and K_2_SiF_6_:Mn^4+^ (KSF) red phosphors were purchased from Shenzhen looking long technology Co., LTD.

### Fabrication of large-area PLTs (15 cm × 25 cm)

Various combination of perovskite powders, 0.0212 g CsBr and 0.0367 g PbBr_2_ (for green-emitting CsPbBr_3_), 0.0168 g CsCl, 0.0177 g PbCl_2_ and 0.005 g CsBr (for blue-emitting CsPb(Cl/Br)_3_), or 0.002 g CsBr, 0.026 g CsI and 0.036 g PbI_2_ (for red-emitting CsPb(Br/I)_3_) were ground with 0.013 g HPβCD for 1 h to obtain perovskite@HPβCD composite powders. Next, the perovskite@HPβCD powder, 150 µl PFOS and 0.17 g PS were mixed together in 1 ml DMF/DMSO mixed solvent (7:3 volume ratio) to prepare the electrospinning ink (Note: this method can be scaled up.). The ink was poured into a syringe (1 mL) fitted with a metallic needle (inner diameter of 0.45 mm). During the process of electrospinning, the syringe was positioned horizontally on a syringe pump, the electrode at the high voltage power supply was clamped to the metal needle tip and ground to an aluminum collector wrapped with aluminum foil (the supporting substrate of PLTs). An applied voltage of 10 kV, a tip-to-collector distance of 10 cm, and a solution flow rate of 1.0 mL h^−1^ was set for weaving the PLTs. The electrospinning apparatus (E02, Foshan Qingzi Precision Measurement and Control Technology Co., Ltd) was enclosed in a Plexiglass box and the electrospinning was carried out at 22-26 °C under 50-60% relative humidity.

### WLEDs fabrication

In total, 5.8 mg of CsPbBr_3_@HPβCD@PFOS fibers were mixed with 5 mg of BaMgAl_10_O_17_:Eu blue-emitted powder and 15 mg of commercial K_2_SiF_6_:Mn^4+^ (KSF) red phosphors. The mixed phosphors were applied to 380 nm purple-emitting InGaN chips (Shenzhen looking long technology co., LTD.) to obtain WLEDs, which operated at a working voltage of 5 V and a current of 10 mA.

### Setup of the dynamic seawater scouring apparatus

A setup of a home-made dynamic seawater scouring apparatus was composed of a peristaltic pump, a 1000 ml beaker and film holder. The CsPbBr_3_@HPβCD@PFOS PLT was set in the film holder under dynamic water scouring, which continuously emitted green light upon UV light irradiation.

### Characterization

The UV-vis absorption (Shimadzu) equipped with an integrating sphere was used to characterize the absorption of CsPbBr_3_, CsPbBr_3_@HPβCD and CsPbBr_3_@HPβCD@PFOS fibers. The morphological features of the samples were characterized using a cold-field emission scanning electron microscope (SEM, Regulus 8230), equipped with a flat-inserted energy spectrum (spatial resolution 10 nm) and YAG back-scattered electron detector (secondary electron mode (SE) and backscattered electron image (BSE) mode can be chosen). The microstructures of the fibers were examined using a transmission electron microscope (TEM, FEI Tecnai G2 F30, 300 kV.) and spherical aberration-corrected transmission electron microscope (JEM-ARM200P, 200 kV) operating at an accelerating voltage of 200 kV. The bright field and photoluminescence (PL) images of PLTs were characterized by a fluorescence microscope (Leica, LSM880) and a confocal laser fluorescence microscope (Zeiss, LSM 710 NLO). The temperature-dependent XRD spectra of the samples were measured on an X-ray diffractometer (Bruker D8 ADVANCE). The 2θ range was 5° to 45°, with a step size of 0.02° and a step rate of 10 s, and the temperature range was from 30 to 250 °C. Room-temperature XRD analysis was also collected (Rigaku D-MAX 2200 VPC). X-ray photoelectron spectroscopy (XPS, ESCALAB 250, Thermo Fisher Scientific) was used to investigate the chemical identity of the samples. The sample surface chemistry was probed with mono Al Kα (1486.6 eV) at 150 W (12 kV, 6 mA). The absolute PLQY values were assessed by a C 9920-02 absolute PL quantum yield measurement system (Hamamatsu Spectral Photometry C9920-02G.). The optical performance of the WLEDs was measured by an OSHP 350 M spectrophotometer with a 50 cm integrating sphere. An Edinburgh Instruments Ltd FLS1000 was used to test steady-state PL, time-resolved PL decay (TRPL), temperature-dependent PL mapping, and temperature-dependent TRPL. The sample was cooled to 150 K firstly, and gradually increased by 30 K until reached 390 K and above. Each temperature point was stable for 10 min. Raman spectra were measured with a Renishaw Raman microscope and spectrometer. The spectral range was adjusted from 100 to 3100 cm^−1^, and the spectral resolution was 1 cm^−1^. All samples were excited with a 785 nm laser beam. Ten cycles of data collection were implemented to reduce the effect of background noise caused by strong fluorescence. The proton nuclear magnetic resonance (^1^H-NMR) characterization was performed on an AVANCE NEO 600 M (Bruker), with DMSO–d_6_ (Merck) used as the reference NMR solvent. The molecular lengths of CsBr and PbBr_2_ were calculated by Chemdraw 19.0. published in 2019.

### Reporting summary

Further information on research design is available in the Nature Portfolio Reporting Summary linked to this article.

## Supplementary information

Supplementary Information

Description of Additional Supplementary Files

Supplementary Movie 1

Supplementary Movie 2

Supplementary Movie 3

## Data Availability

The source data generated in this study are provided in the ‘Source Data’ file. Source data are provided with this paper.

## Source data

Source data
