# Supplementary material for: Large-area waterproof and durable perovskite luminescent textiles
Source: Nat Commun. 2023 Jan 16;14:234. doi: 10.1038/s41467-023-35830-8 (PMC9842651; doi:10.1038/s41467-023-35830-8)
Supplement: Supplementary file 1 — Supplementary Information [file 41467_2023_35830_MOESM1_ESM.pdf]

Supplementary materials for

Large-area waterproof and durable perovskite luminescent textiles

Tian Tian[1 #], Meifang Yang[1 #], Yuxuan Fang[1], Shuo Zhang[1], Yuxin Chen[2], Lianzhou Wang[3]*, Wu-Qiang Wu[1]*

[1]MOE Key Laboratory of Bioinorganic and Synthetic Chemistry, Lehn Institute of Functional Materials, School of Chemistry, Sun Yat-sen University; Guangzhou 510006, P. R. China

[2]Instrumental Analysis and Research Center, Sun Yat-sen University; Guangzhou 510275, P. R. China

[3]Nanomaterials Centre, School of Chemical Engineering and Australian Institute for Bioengineering and Nanotechnology, The University of Queensland; Brisbane, QLD 4072, Australia.

[#]These authors contributed equally to this work.

*Corresponding author. Email: l.wang@uq.edu.au, wuwq36@mail.sysu.edu.cn

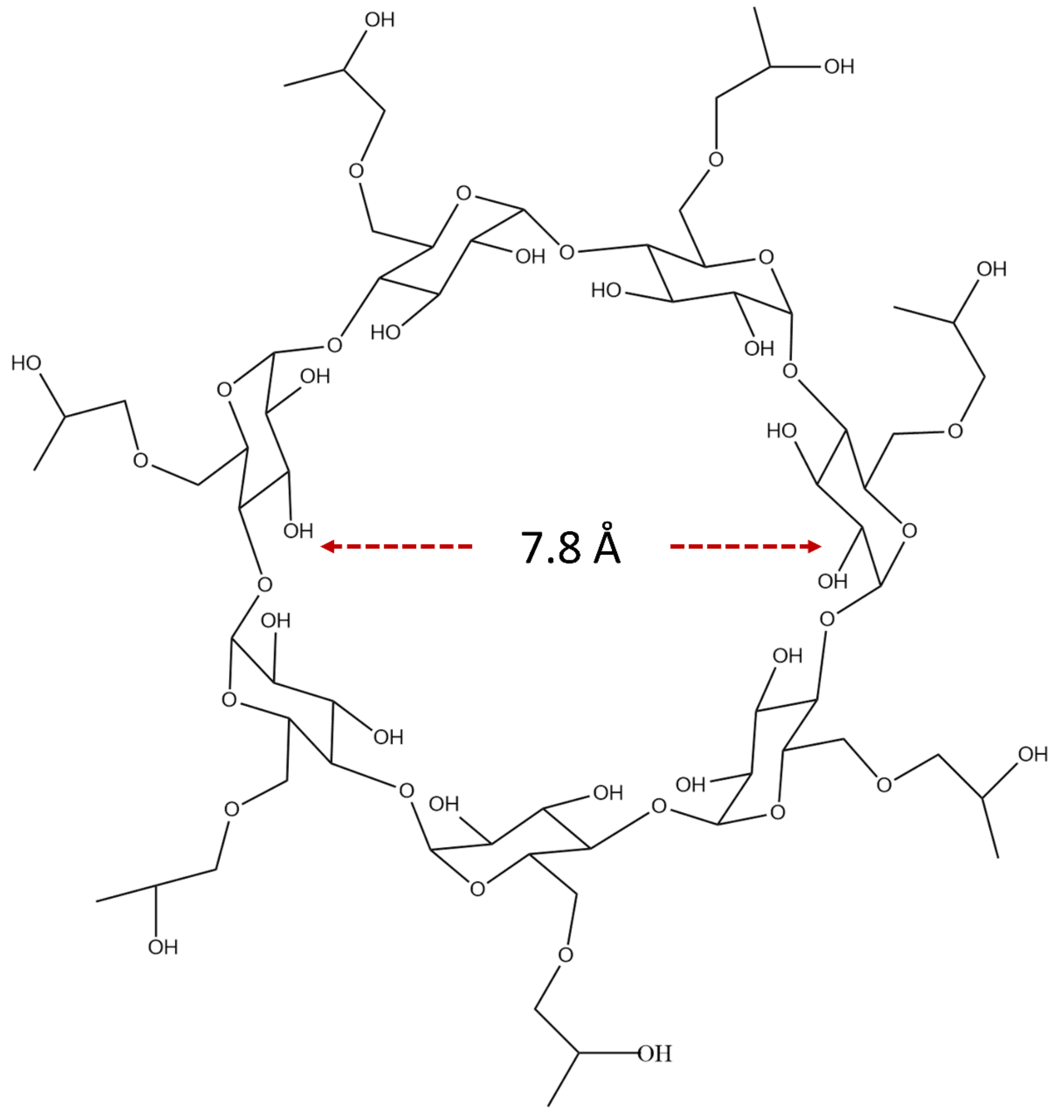

Supplementary Fig. 1 The molecular structure of HPβCD.

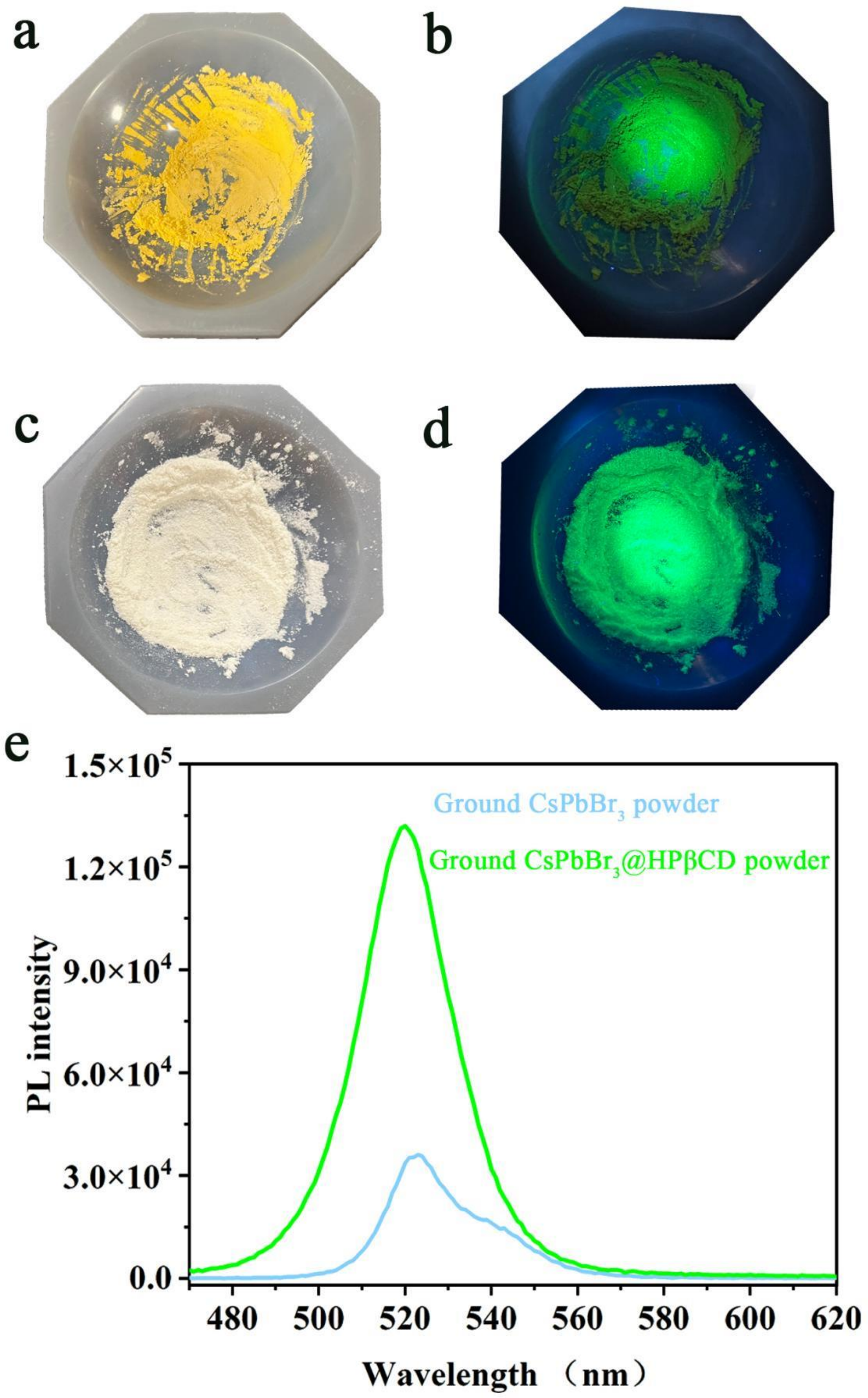

Supplementary Fig. 2. Ground CsBr and $PbBr_2$ blends with or without HPβCD powder addition and the corresponding PL intensity spectra. Photographs of ground powder under natural light and UV light irradiation: (a, b) $CsPbBr_3$, (c, d) $CsPbBr_3$@HPβCD. (e) PL spectra of ground $CsPbBr_3$ powder and $CsPbBr_3$@HPβCD powder.

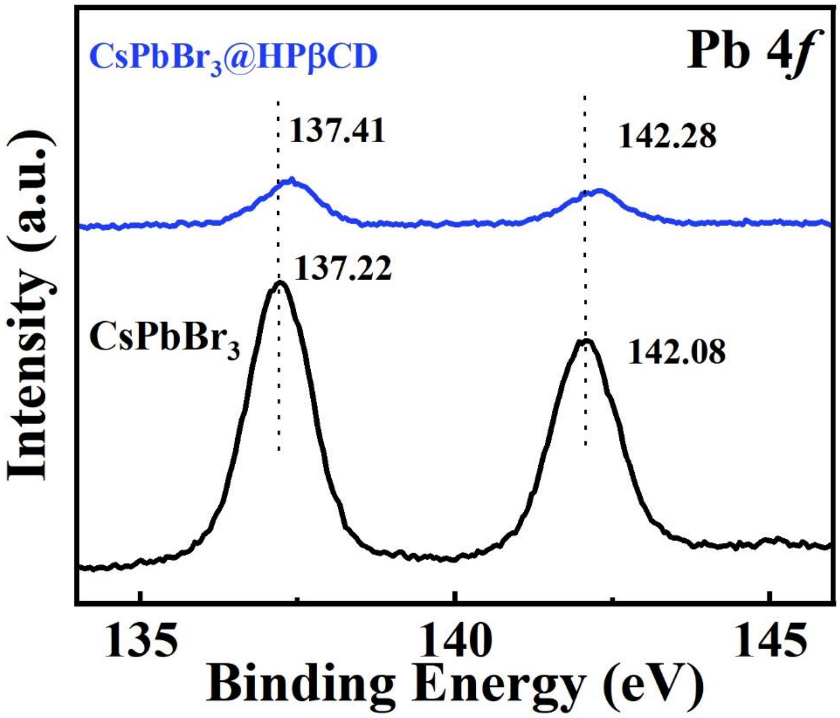

Supplementary Fig. 3. XPS spectra of Pb 4f signal of $CsPbBr_3$ and $CsPbBr_3$@HPβCD samples.

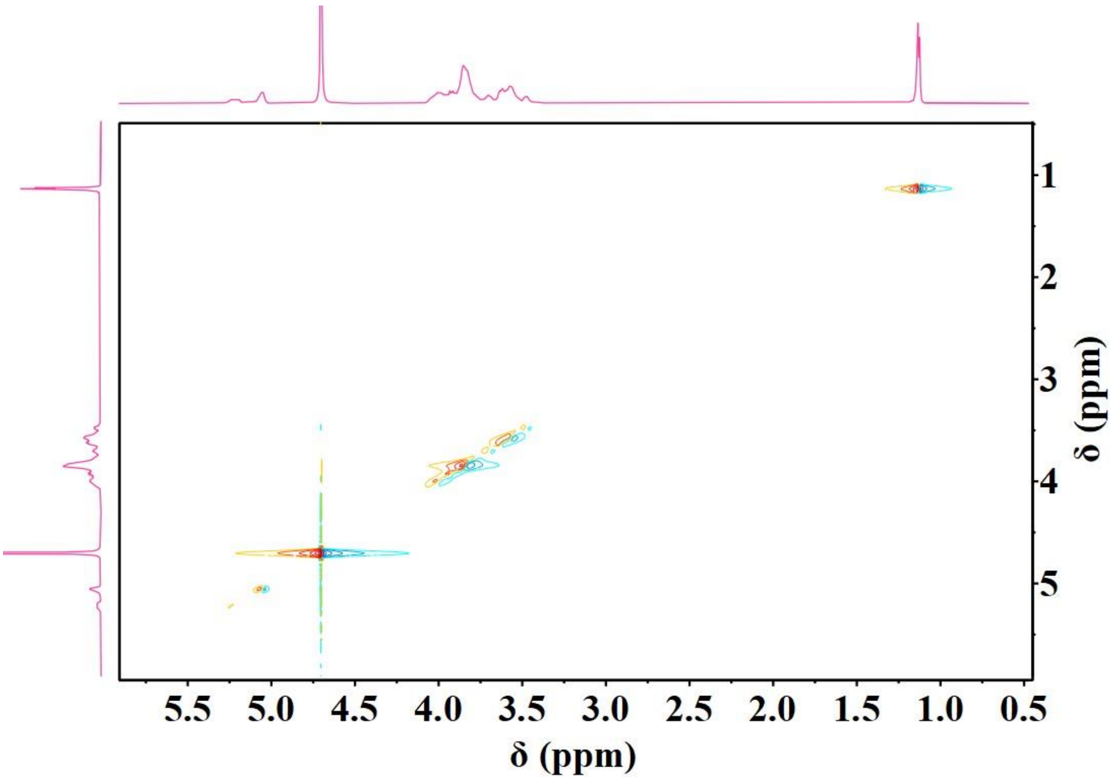

Supplementary Fig. 4. 2D ^{1}H–^{1}H NOESY spectrum of HPβCD.

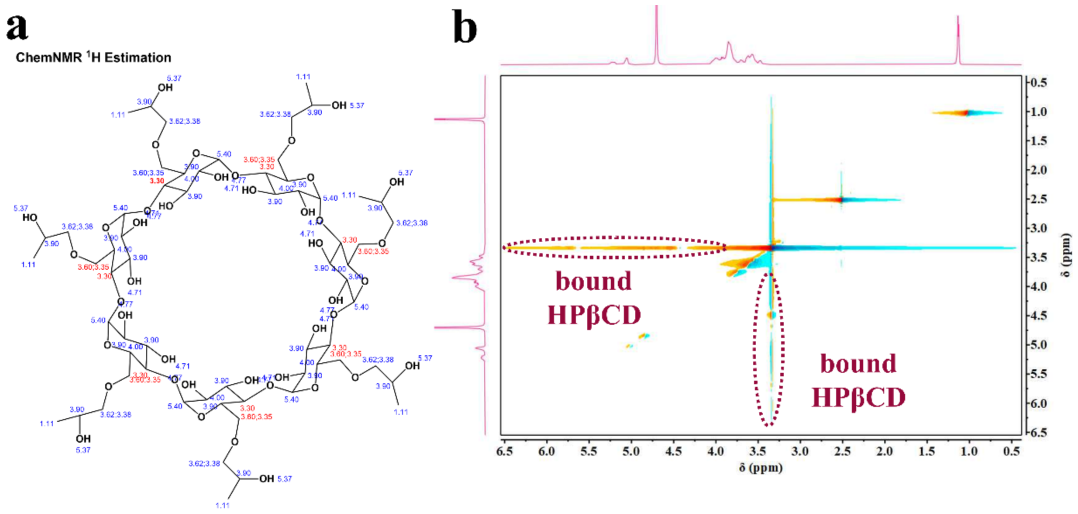

Supplementary Fig. 5. The ^{1}HNMR of HPβCD molecule and NOESY spectrum. (a) The ^{1}HNMR of HPβCD molecule estimated by ChemDraw 19.0 software. (b) NOESY spectrum of $CsPbBr_3$@HPβCD composites dispersed in deuterated dimethyl sulfoxide (D_6MSO).

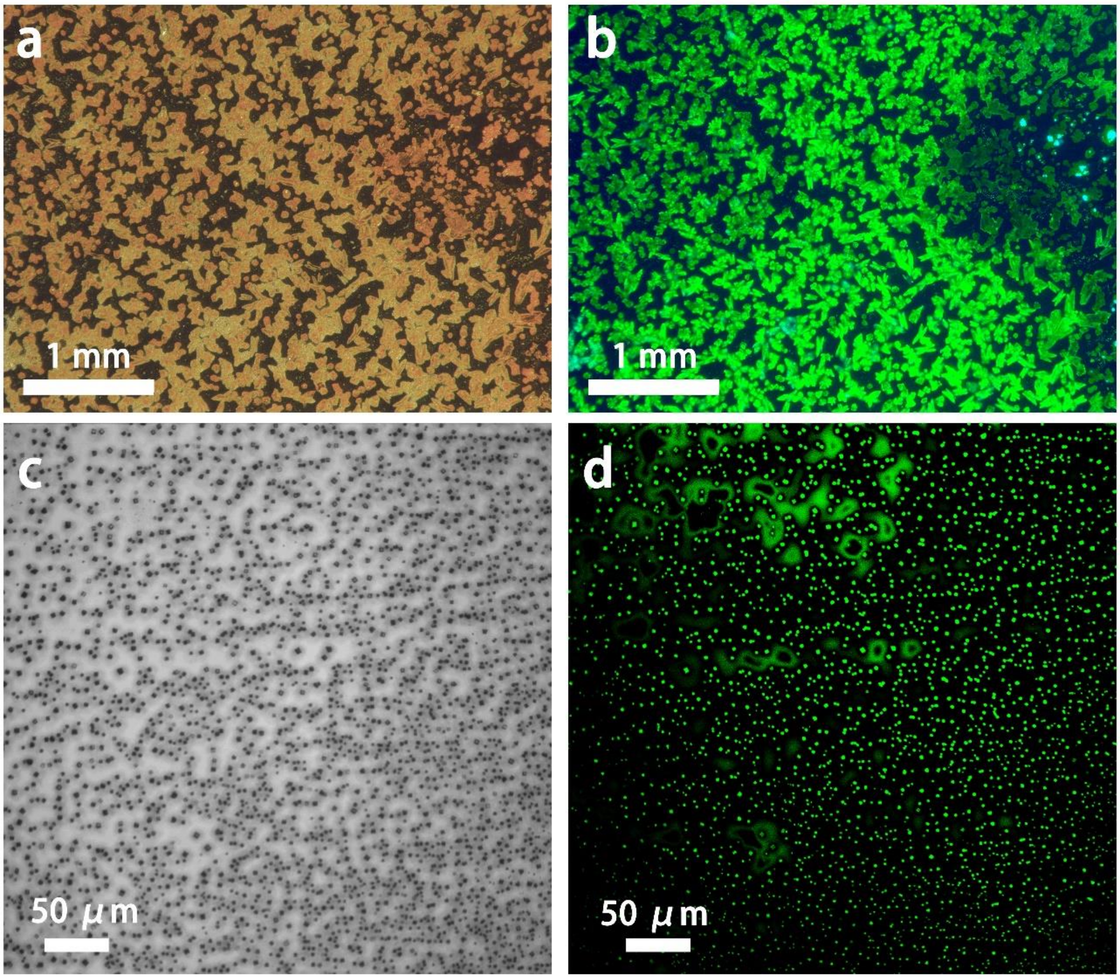

Supplementary Fig. 6. Bright-field and fluorescence microscopy images. The bright field and corresponding fluorescence images of (a, b) $CsPbBr_3$ powders characterized by fluorescence microscopy and (c, d) $CsPbBr_3$@HPβCD composited powders characterized by confocal laser fluorescence microscopy.

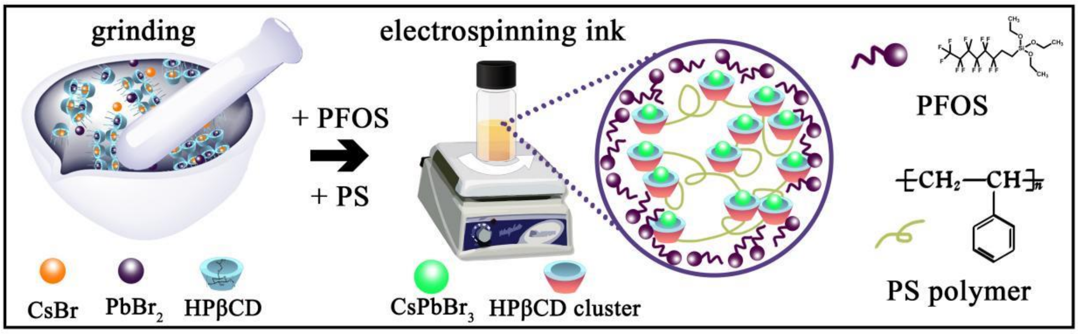

Supplementary Fig. 7. Schematic illustration of the electrospinning ink preparation.

a b

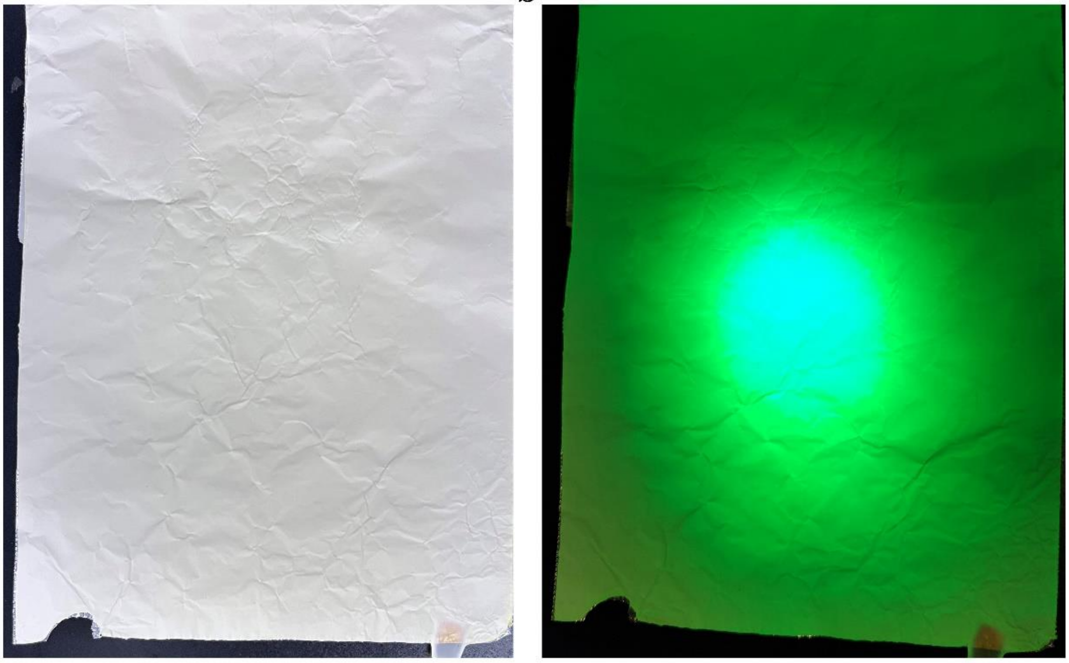

Supplementary Fig. 8. The PLT fabricated with electrospinning ink via directly mixing CsBr, $PbBr_2$, HPβCD, PS and PFOS ingredients: (a) under natural light and (b) under UV light.

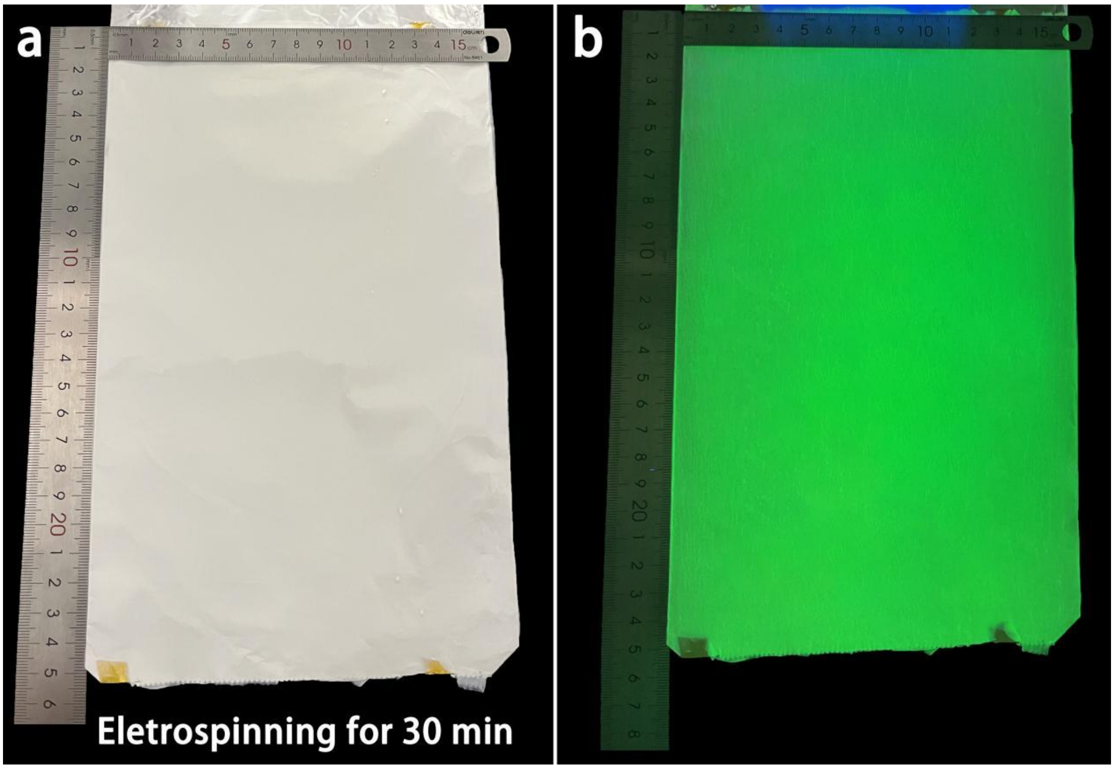

Supplementary Fig. 9. Electrospinning a 25 cm × 15 cm PLT within 30 min. Photographs of a PLT prepared via electrospinning for 30 min under (a) natural light and (b) UV light.

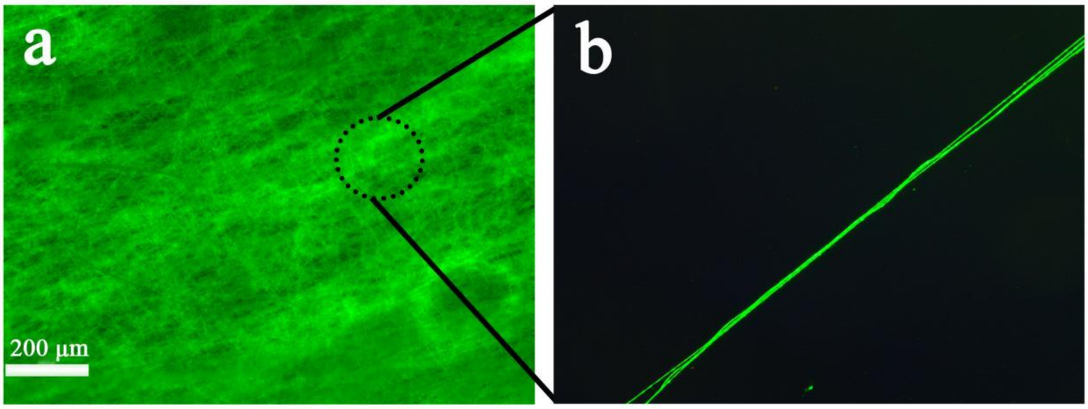

Supplementary Fig. 10. Fluorescence images of fibrous membrane and individual fiber. Fluorescence images of (a) as-prepared $CsPbBr_3$@HPβCD@PFOS fibrous membrane and (b) an individual fiber.

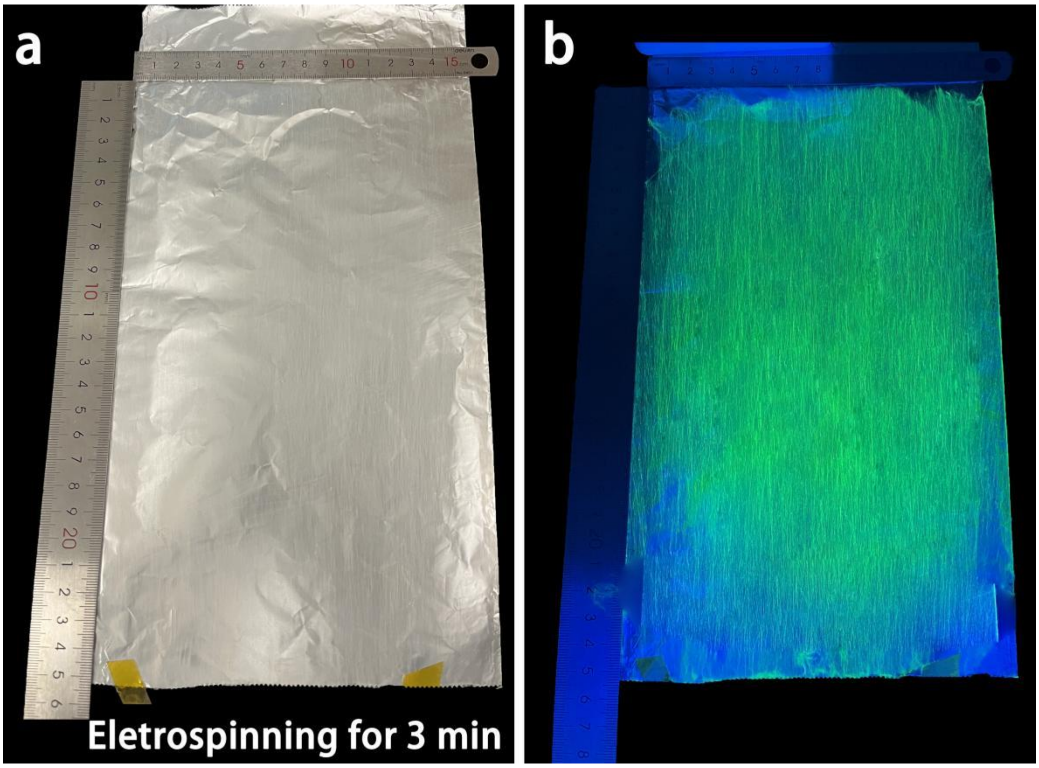

Supplementary Fig. 11. Electrospinning a 25 cm × 15 cm PLT within 3 min. Photograph of the PLT prepared via electrospinning for 3 min under (a) natural light and (b) UV light.

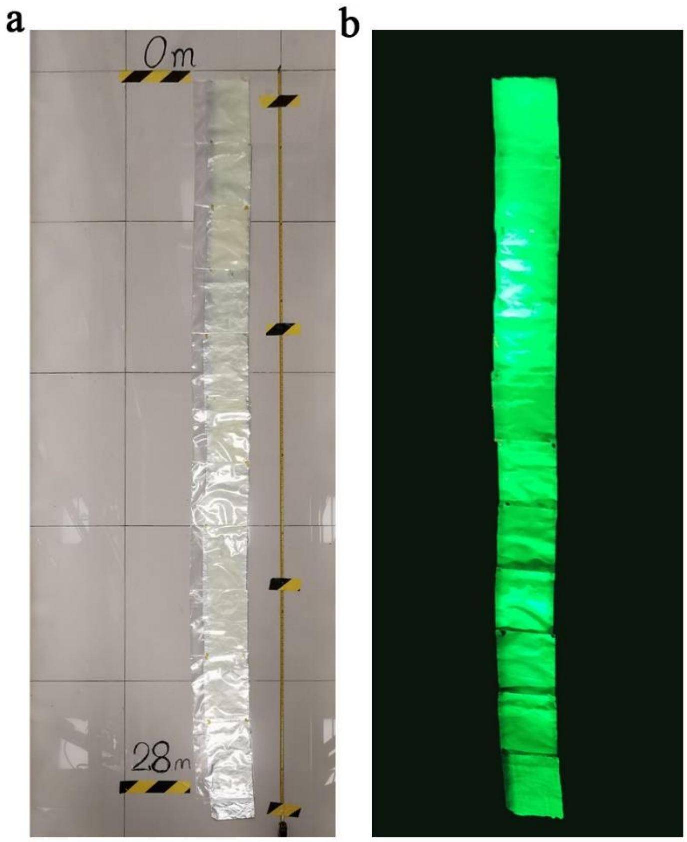

Supplementary Fig. 12. Demonstration of a 2.8 m PLT (280 cm × 15 cm) under (a) natural light and (b) UV light irradiation.

Supplementary Table 1. Cost evaluation of the luminous textile (280 cm ×15 cm, equal to an area of 0.42 m^2).

	**Ingredients**	**Unit price (USD)**	**Usage amount**	**Price (USD)**	
**PLTs fabrication**	CsBr	34.65/50 g	0.170 g	0.12	Cost: **0.05 cents m^{-2}**
	$PbBr_2$	9.31/25 g	0.294 g	0.11	
	HPβCD	16.38/100 g	0.104 g	0.02	
	PFOS	33.86/25 mL	1.2 mL	1.63	
	DMF	3.15/500 ml	5.6 ml	0.04	
	DMSO	6.93/100 ml	2.4 ml	0.17	
	PS	11.09/500 g	1.22 g	0.03	
**Total price**				2.12	

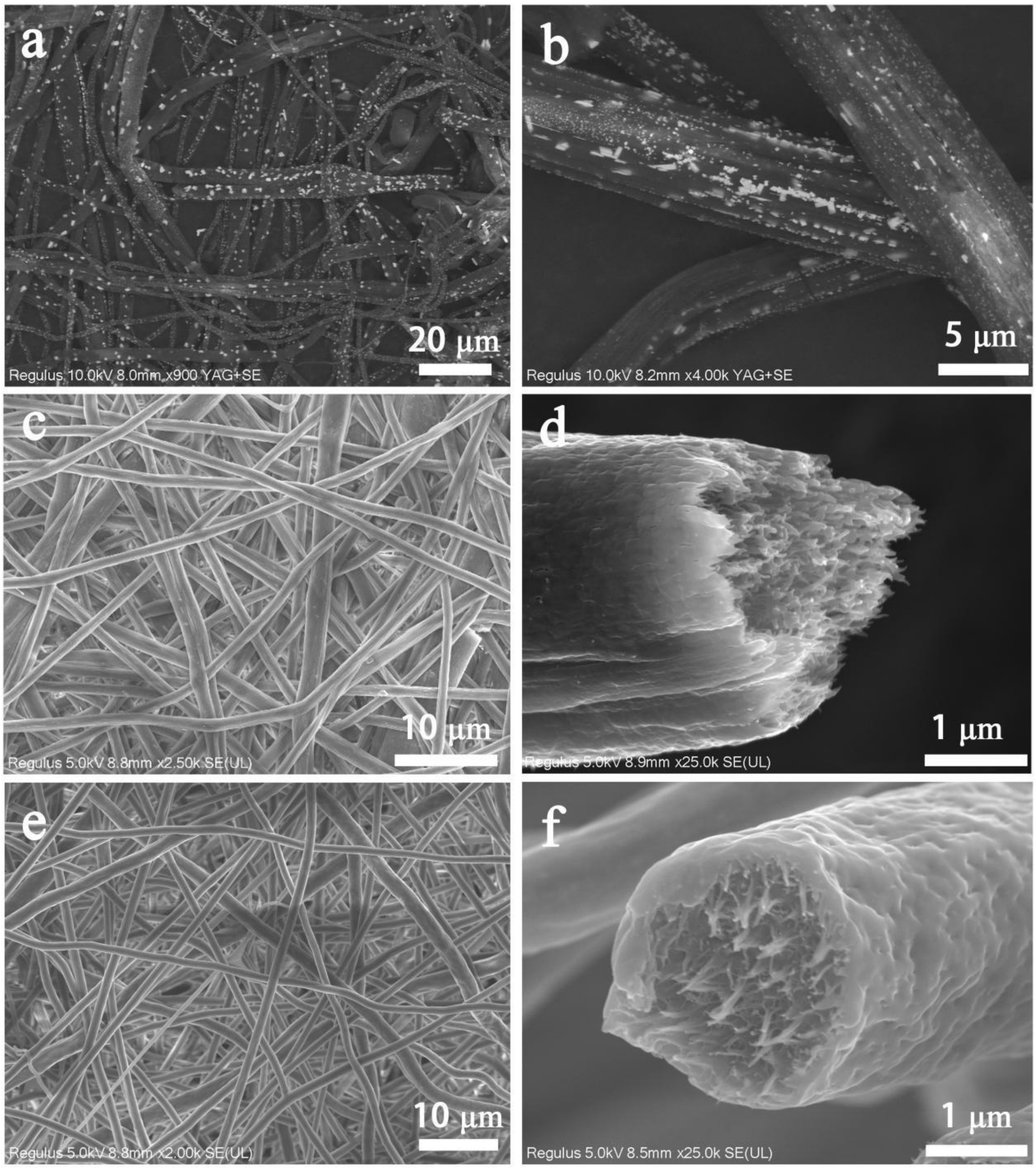

Supplementary Fig. 13. Morphological structures of different PLTs. SEM images of (a, b) $CsPbBr_3$ fibers, (c, d) $CsPbBr_3$@HPβCD fibers and (e, f) $CsPbBr_3$@HPβCD@PFOS fibers.

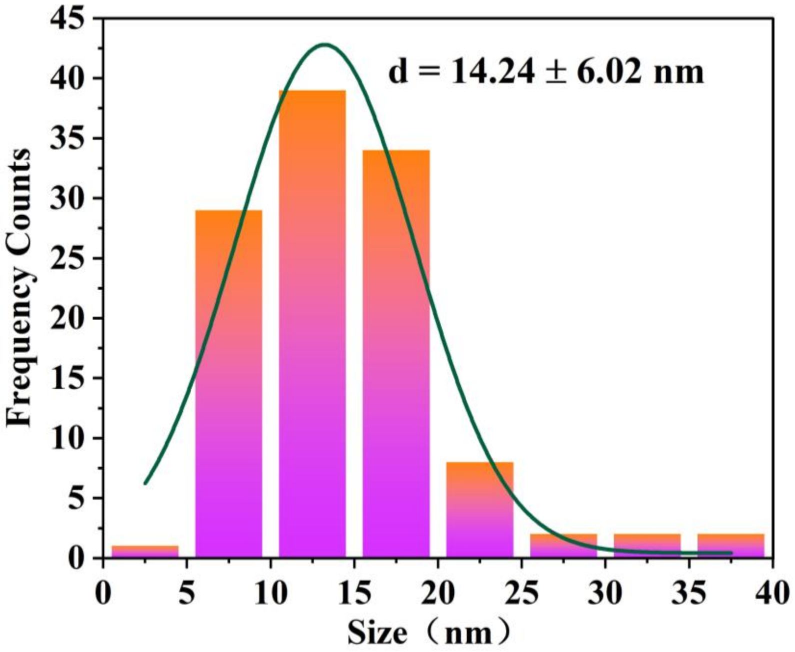

Supplementary Fig. 14. The statistic of size distribution of $CsPbBr_3$ NCs in the $CsPbBr_3$@HPβCD@PFOS fiber.

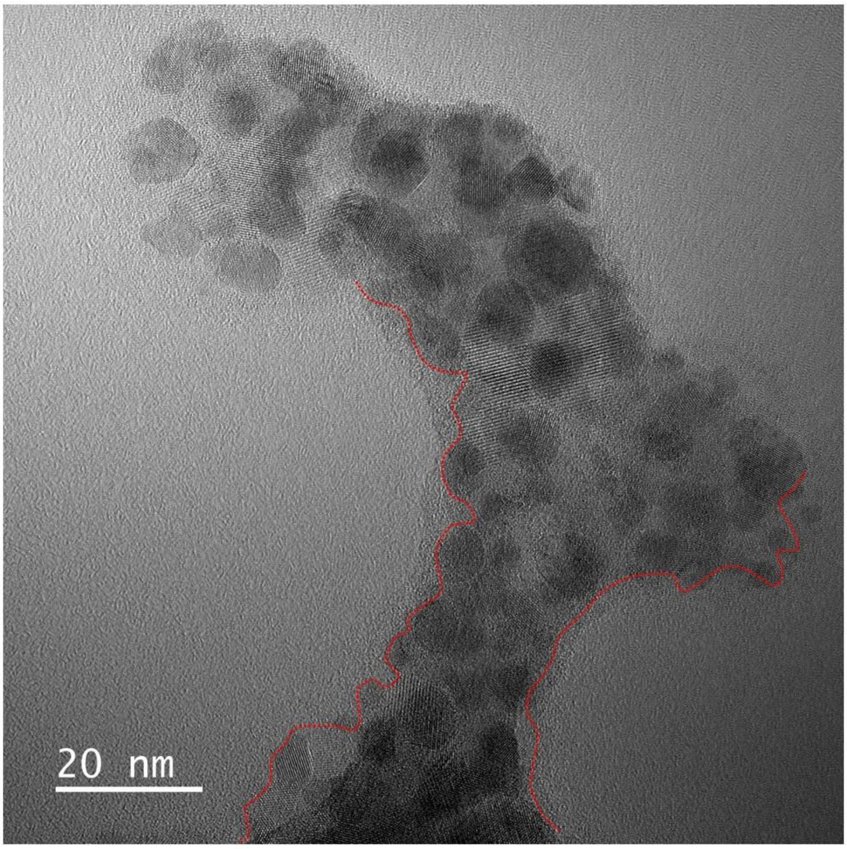

Supplementary Fig. 15. The HRTEM image of $CsPbBr_3$@HPβCD.

Note: the $CsPbBr_3$@HPβCD composited nanocrystals were prepared by dissolving the $CsPbBr_3$@HPβCD fibers in chlorobenzene (CB) solution followed by ultrasonic dispersion.

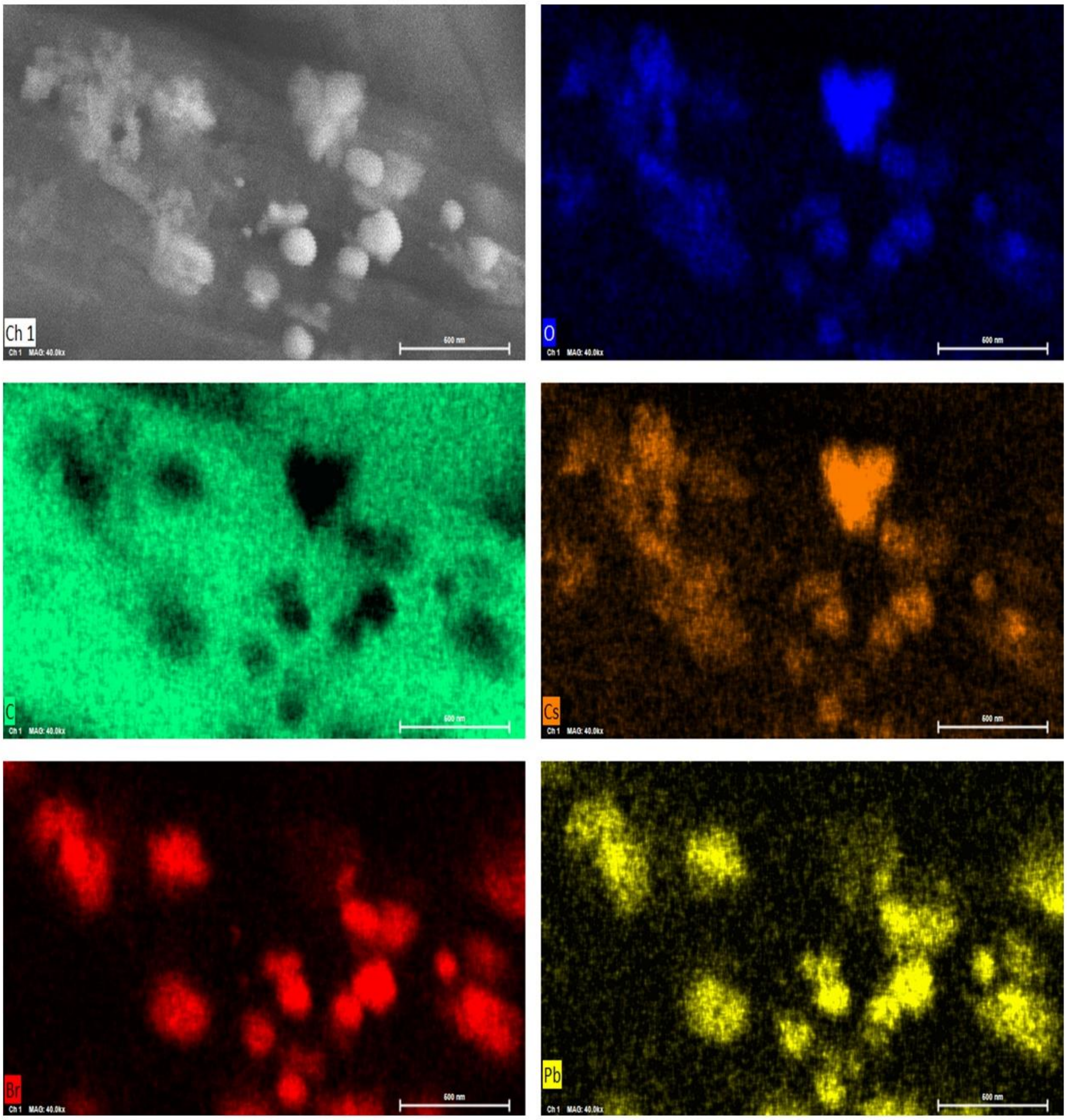

Supplementary Fig. 16. The SEM elemental mapping of $CsPbBr_3$ fibers.

Supplementary Table 2. The element percentage of $CsPbBr_3$ fibers.

Element	At.No.	Mass Norm. [%]	Atom [%]	Abs.error [%]
**C**	6	59.07	89.90	1.31
**Cs**	55	22.27	3.06	0.60
**Br**	35	7.34	1.68	0.10
**Pb**	82	7.19	0.63	0.10
**O**	8	4.13	4.72	0.13
**Total**		100.00	100.00	

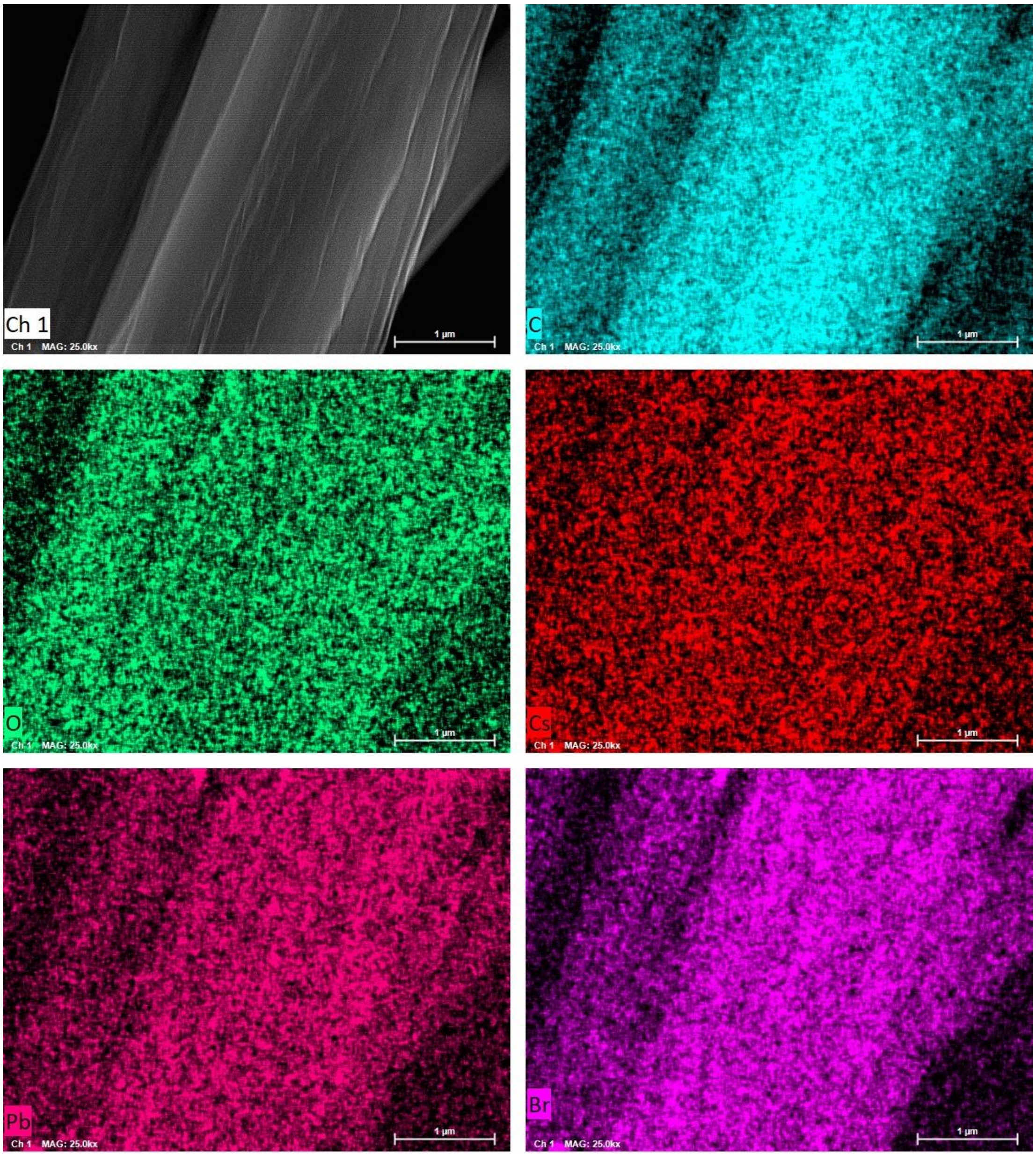

Supplementary Fig. 17. The SEM elemental mapping of $CsPbBr_3$@HPβCD fibers.

Supplementary Table 3. The element percentage of $CsPbBr_3$@HPβCD fibers.

Element	At.No.	Mass Norm.[%]	Atom [%]	Abs.error [%]
**C**	6	86.08	90.91	8.77
**Cs**	55	0.63	0.06	0.05
**Br**	35	1.36	0.22	0.09
**Pb**	82	0.88	0.05	0.06
**O**	8	11.06	8.77	1.30
**Total**		100.00	100.00	

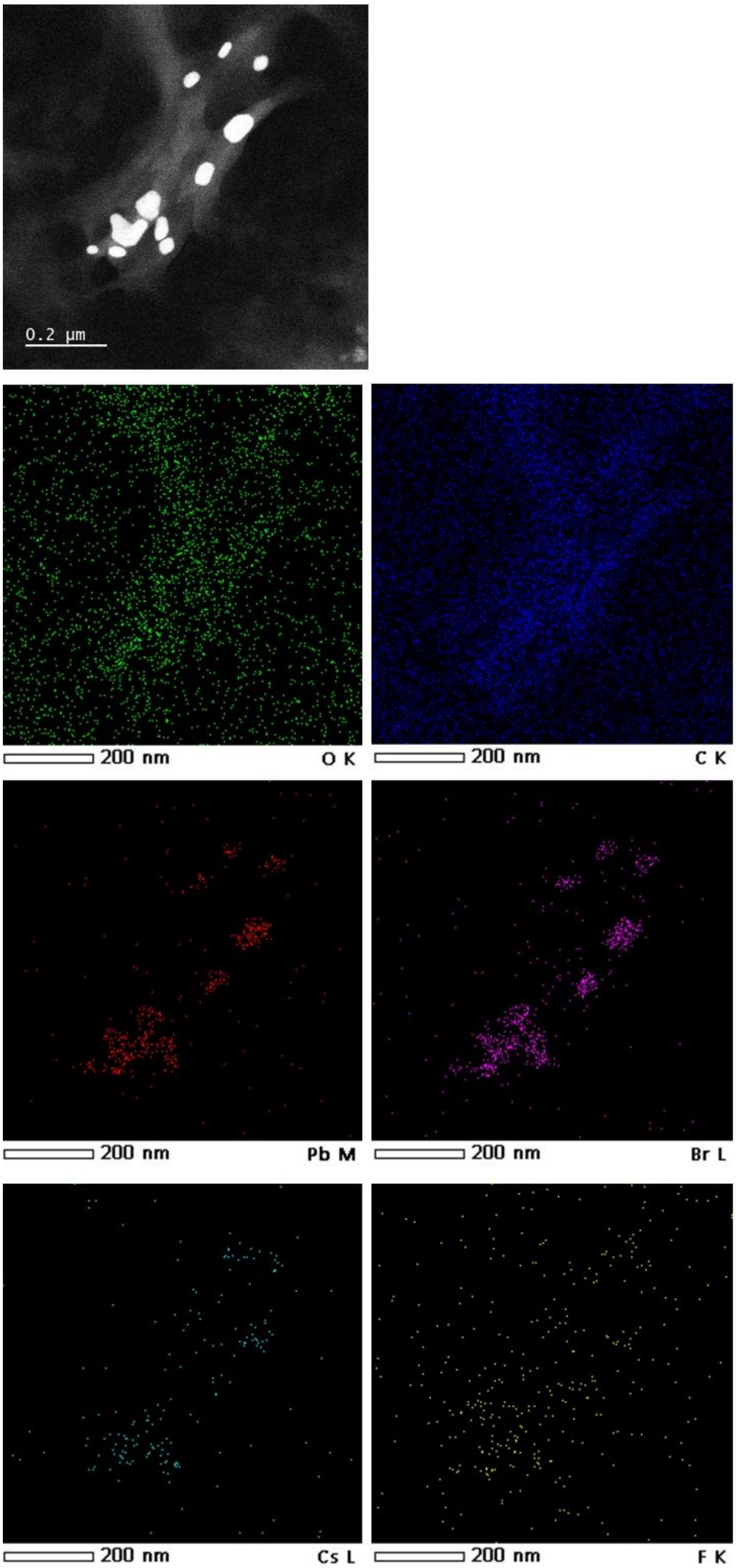

Supplementary Fig. 18. The HRTEM elemental mapping of $CsPbBr_3$ nanocrystals in $CsPbBr_3$@HPβCD@PFOS fibers.

Supplementary Table 4. The element percentage of $CsPbBr_3$@HPβCD@PFOS fibers.

Element	At.No.	Mass Norm.[%]	Atom [%]	Abs.error [%]
**C**	6	88.22	93.46	0.67
**Cs**	55	0.52	0.05	0.08
**Br**	35	2.18	0.35	0.20
**Pb**	82	1.45	0.09	0.17
**O**	8	7.51	5.97	0.23
**F**	9	0.13	0.08	0.03
**Total**		100.00	100.00	

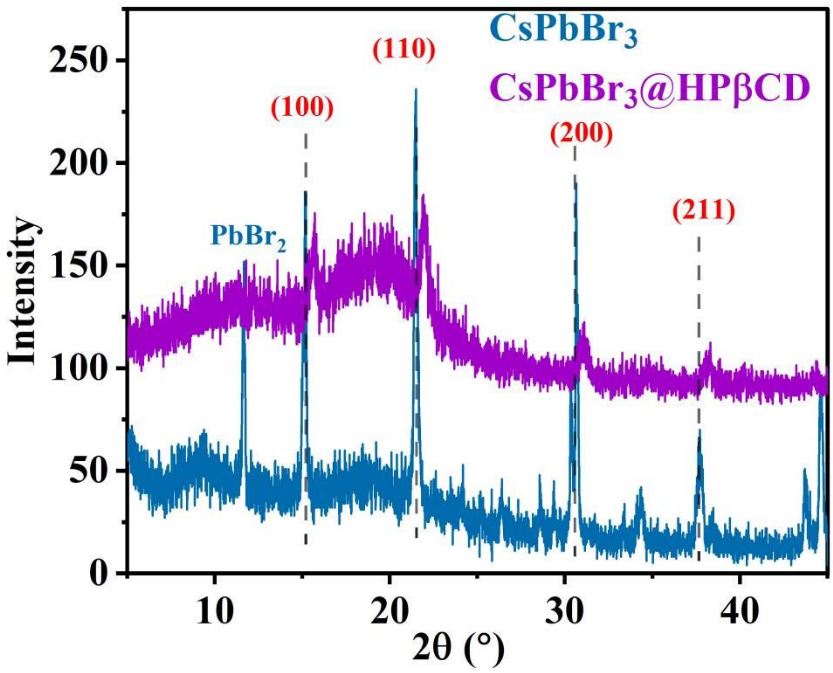

Supplementary Fig. 19. XRD patterns of different PLTs. XRD patterns of $CsPbBr_3$ and $CsPbBr_3$@HPβCD fibers.

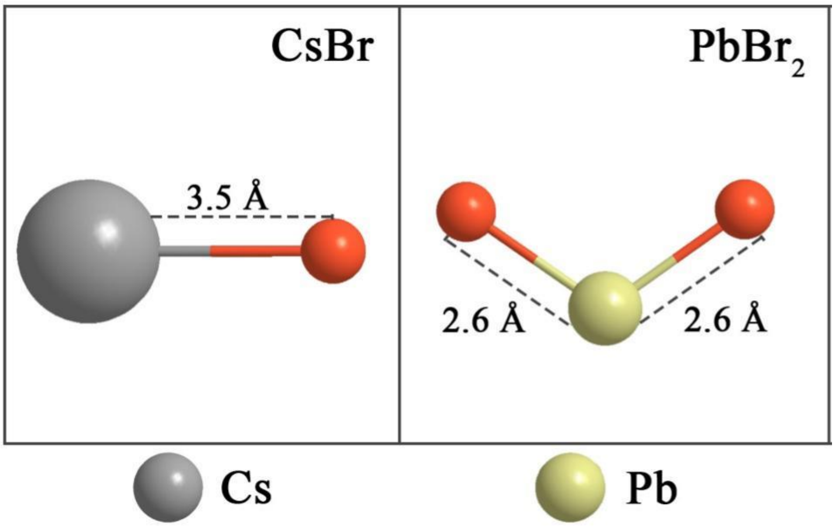

Supplementary Fig. 20. The calculated molecular lengths of CsBr and $PbBr_2$.

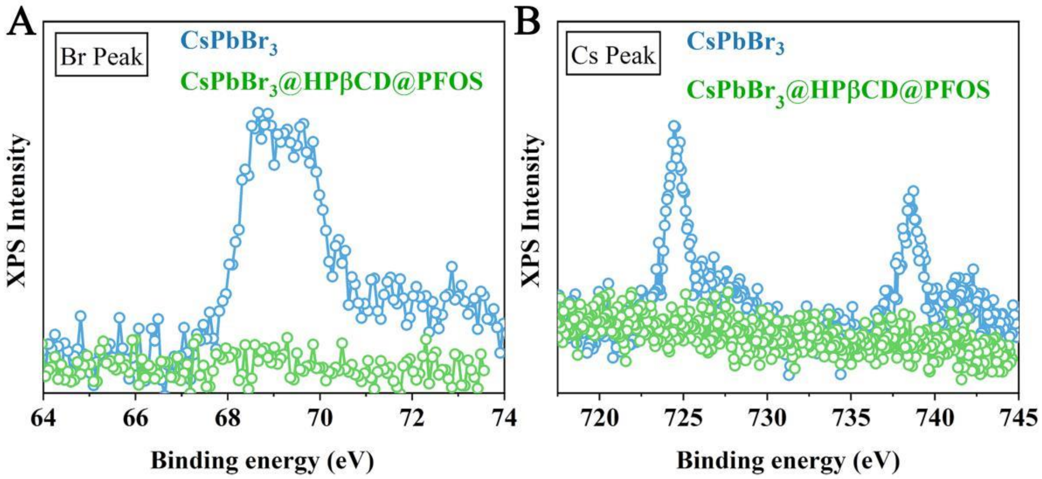

Supplementary Fig. 21. X-ray photoelectron spectroscopy (XPS) of $CsPbBr_3$ and $CsPbBr_3$@HPβCD@PFOS fibers. XPS spectra of the (a) Br signal and (b) Cs signal of the $CsPbBr_3$ fibers and $CsPbBr_3$@HPβCD@PFOS fibers.

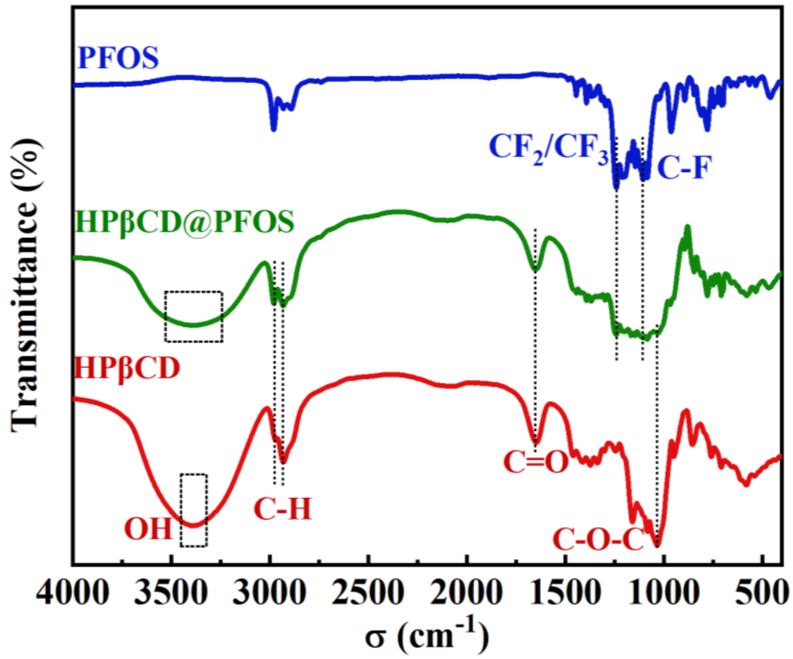

Supplementary Fig. 22. FTIR spectra of HPβCD, PFOS and HPβCD@PFOS.

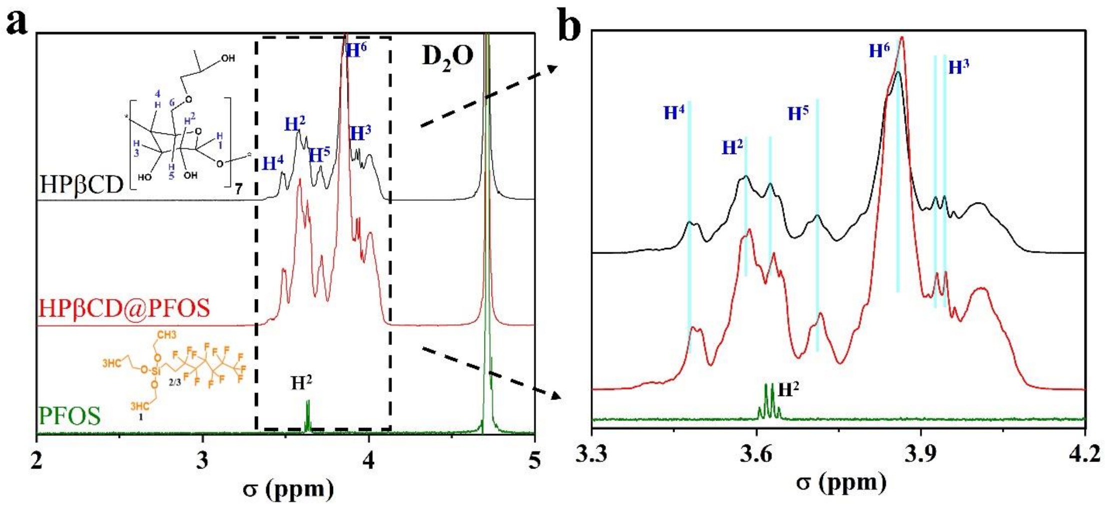

Supplementary Fig. 23. The ^{1}HNMR spectra of HPβCD, PFOS and HPβCD@PFOS (a), and its enlarged view as indicated (b).

Supplementary Table 5. The ^{1}H NMR positions of HPβCD and HPβCD@PFOS.

Proton	HPβCD (ppm)	HPβCD@PFOS (ppm)
H^2	3.6248	3.6321
H^3	3.9265	3.9293
H^4	3.4777	3.4835
H^5	3.7106	3.7165
H^6	3.8584	3.8654

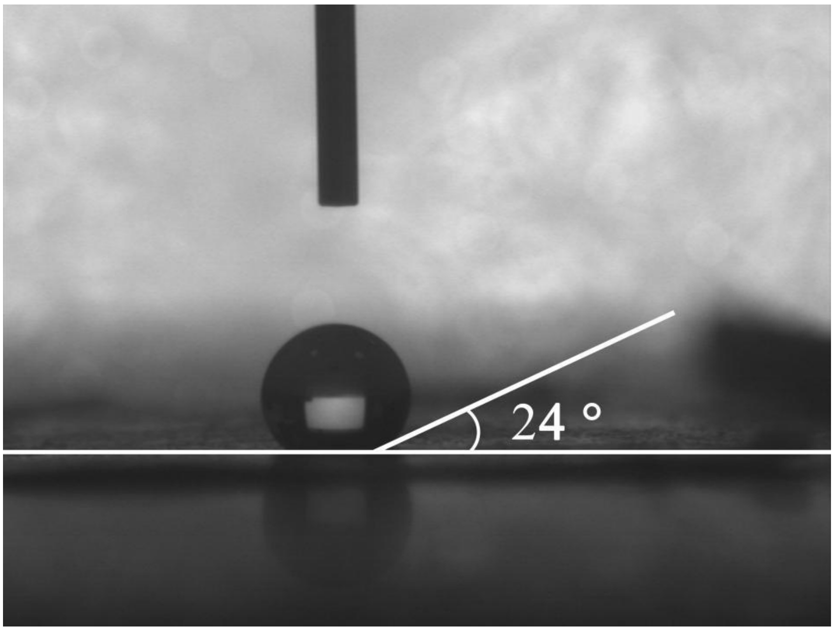

Supplementary Fig. 24. Surface wettability characterization. Contact angle testing of a water drop on the PLT.

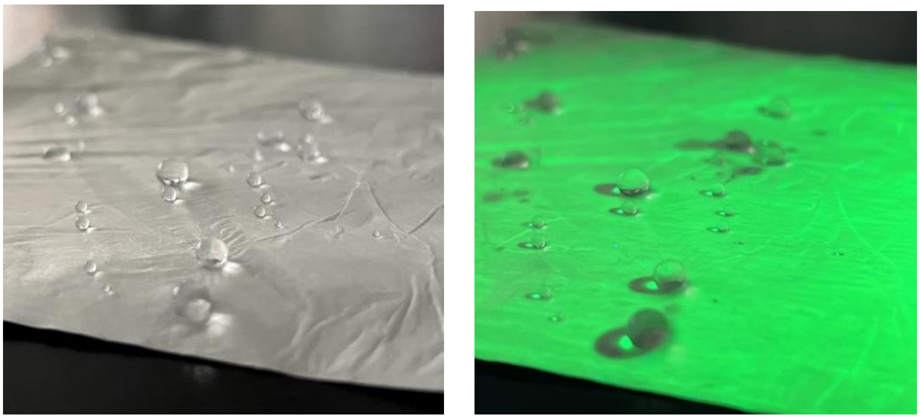

Supplementary Fig. 25. Surface hydrophobicity of the PLT. Images of water droplets on the $CsPbBr_3$@HPβCD@PFOS fibrous membrane under natural light (left) and UV light (right).

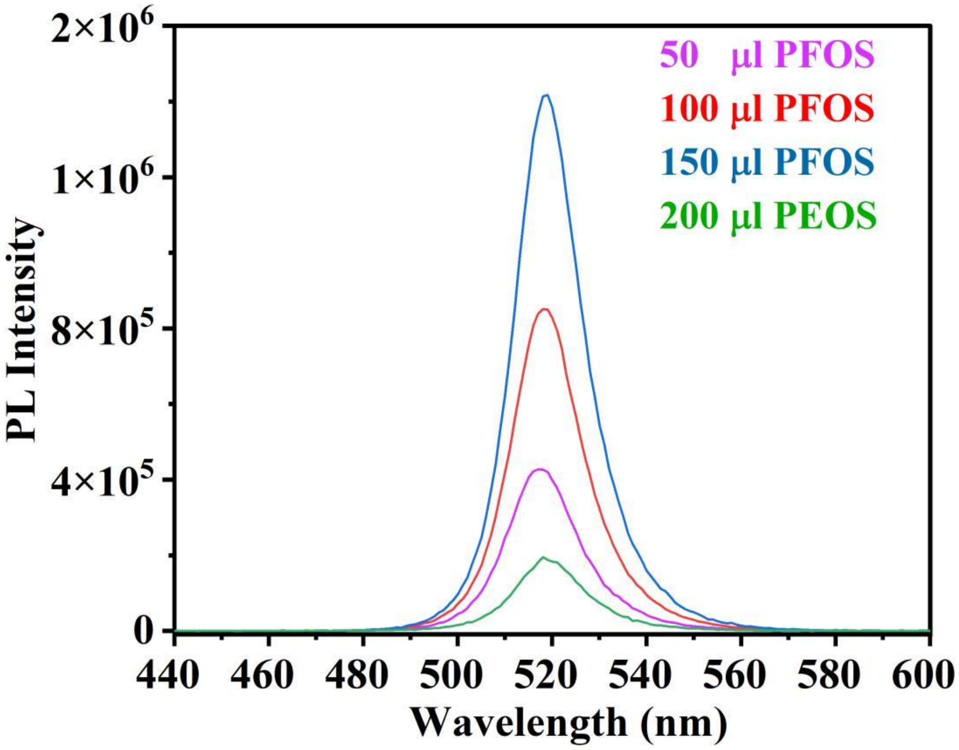

Supplementary Fig. 26. Optimization of PFOS content. PL intensity spectra of the $CsPbBr_3$@HPβCD@PFOS sample fabricated with different PFOS contents.

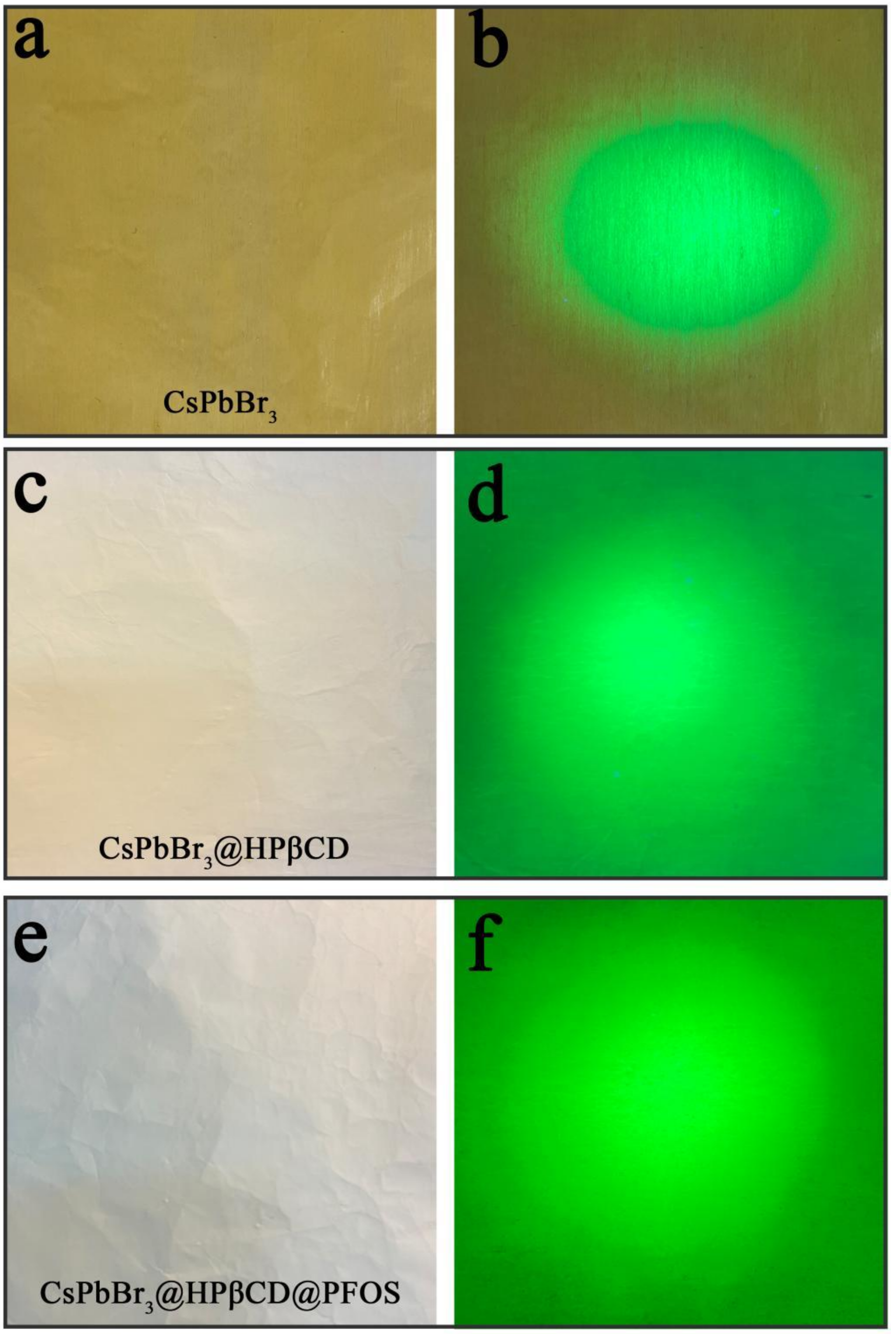

Supplementary Fig. 27. Luminescent images of different PLTs under natural light (left) and UV light (365 nm, right): (a, b) $CsPbBr_3$, (c, d) $CsPbBr_3$@HPβCD and (e, f) $CsPbBr_3$@HPβCD@PFOS.

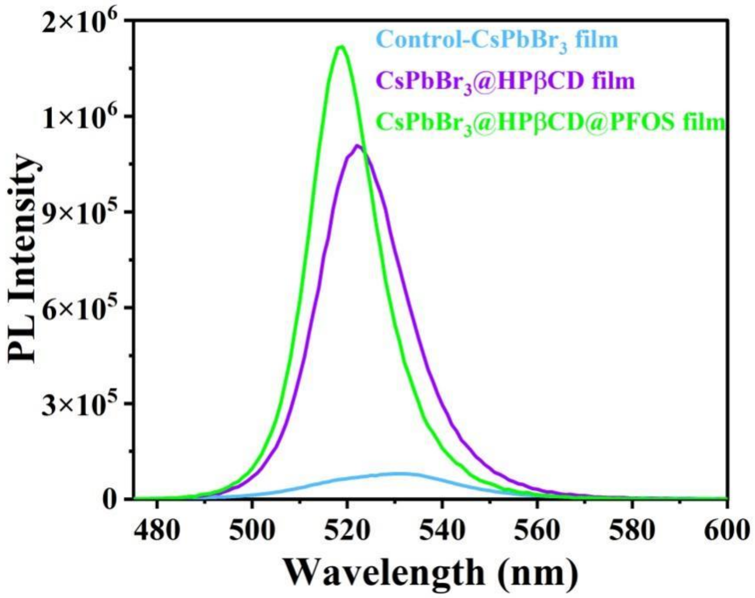

Supplementary Fig. 28. PL intensities of different PLTs. The PL intensity comparison of $CsPbBr_3$, $CsPbBr_3$@HPβCD and $CsPbBr_3$@HPβCD@PFOS fibers at the same excitation slit.

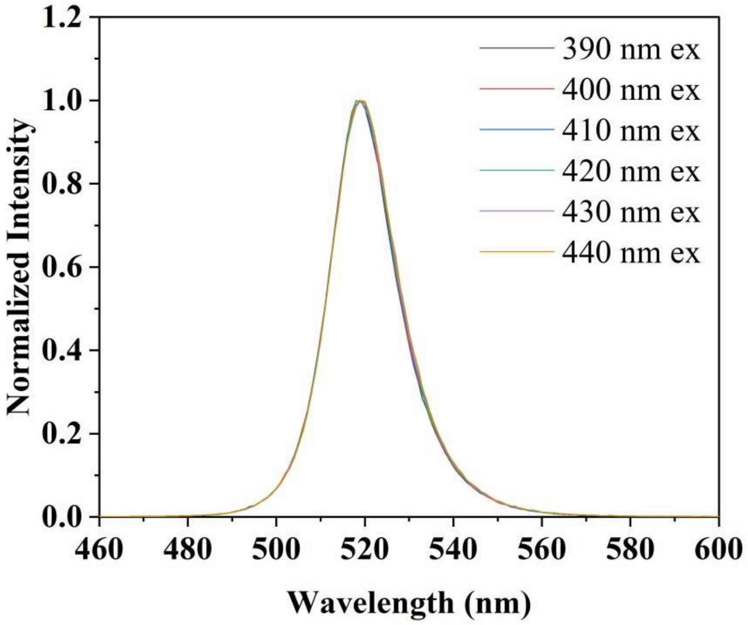

Supplementary Fig. 29. The excitation wavelength-dependent PL spectra of $CsPbBr_3$@HPβCD@PFOS fibers.

Supplementary Table 6. The parameters of average lifetime (τ_a), PLQY, radiative recombination rate and non-radiative recombination rate for different fibers at 300 K.

Samples	τ_a ns	PLQY %	κ_{rad} $10^6/s^1$ *	κ_{non} $10^6/s^1$ *
$CsPbBr_3$	9.25	2.15	2.32	10.81
$CsPbBr_3$@HPβCD	93.85	33.89	3.84	6.82
$CsPbBr_3$@HPβCD@PFOS	52.80	49.70	9.41	9.53

* The radiative recombination and non-radiative recombination rate are calculated based on the following equations (1):

$$PLQY = \kappa_{rad}/(\kappa_{rad+}\kappa_{non}), \quad \tau_a = 1/(\kappa_{rad+}\kappa_{non}) \qquad (1)$$

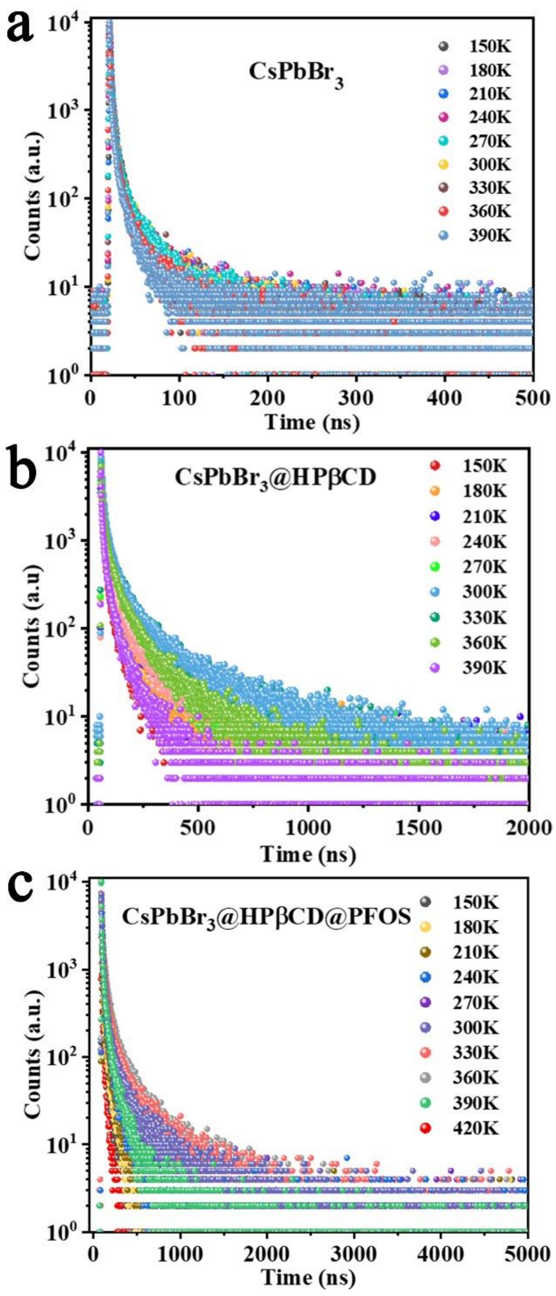

Supplementary Fig. 30. Temperature-dependent time-resolved photoluminescence (TRPL) characterization. (a) $CsPbBr_3$ fibers, (b) $CsPbBr_3$@HPβCD fibers and (c) $CsPbBr_3$@HPβCD@PFOS fibers.

Supplementary Table 7. The PL lifetime decay parameters of $CsPbBr_3$ fibers at different temperatures.

**Temperature (K)**	**τ_1 (ns)**	**A_1 %**	**τ_2 (ns)**	**A_2 %**	**τ_3 (ns)**	**A_3 %**	**τ_{av}* (ns)**
**150**	0.98	42.71	5.85	46.35	38.35	10.94	7.33
**180**	1.00	40.61	4.96	47.33	41.97	12.06	7.82
**210**	0.92	35.70	4.41	47.83	37.87	16.47	8.67
**240**	0.87	38.86	5.26	42.44	42.05	17.70	10.02
**270**	0.97	47.67	5.87	37.00	42.93	15.33	9.22
**300**	0.93	47.38	5.78	36.44	41.49	16.18	9.26
**330**	0.95	50.05	6.41	35.25	40.51	14.70	8.69
**360**	0.86	55.11	5.45	32.26	32.38	12.63	6.32
**390**	0.82	57.93	5.40	31.17	29.02	10.89	5.32

*The average lifetime (τ_{av}) can be calculated as the function (2):

$$\tau_{av} = \frac{A_1\tau_1^2+A_2\tau_2^2+A_3\tau_3^2}{A_1\tau_1+A_2\tau_2+A_3\tau_3} \quad (2)$$

Supplementary Table 8. The PL lifetime decay parameters of $CsPbBr_3$@HPβCD fibers at different temperatures.

**Temperature (K)**	**τ_1 (ns)**	**A_1 %**	**τ_2 (ns)**	**A_2 %**	**τ_3 (ns)**	**A_3 %**	**τ_{av}* (ns)**
**150**	3.73	44.85	20.48	42.08	147.01	13.07	29.51
**180**	4.46	45.05	24.65	40.26	179.10	14.69	38.24
**210**	4.85	43.45	26.52	40.14	185.56	16.41	43.20
**240**	5.44	36.96	29.71	44.04	209.02	19.00	54.81
**270**	7.06	34.48	38.94	44.76	237.94	20.76	69.27
**300**	7.18	26.79	44.29	45.67	260.39	27.54	93.85
**330**	6.36	21.23	44.51	47.64	232.00	31.14	94.78
**360**	4.49	20.63	34.16	49.06	165.64	30.32	67.90
**390**	2.66	26.88	17.80	48.35	84.18	24.77	30.16

Supplementary Table 9. The PL lifetime decay parameters of $CsPbBr_3$ @HPβCD@PFOS fibers at different temperatures.

Temperature (K)	τ_1 (ns)	A_1 %	τ_2 (ns)	A_2 %	τ_3 (ns)	A_3 %	τ_{av}* (ns)
**150**	2.15	48.15	12.08	35.91	68.71	15.94	16.33
**180**	2.72	48.89	10.00	28.16	68.29	22.96	19.82
**210**	3.91	41.16	21.04	42.16	105.98	16.68	28.15
**240**	4.85	41.42	27.45	42.80	145.95	15.77	36.78
**270**	5.72	40.74	31.16	43.06	179.33	16.20	44.80
**300**	7.07	40.91	39.52	42.54	200.00	16.55	52.80
**330**	7.59	32.62	41.77	42.04	257.99	25.34	85.41
**360**	7.24	27.09	43.70	45.79	252.70	27.12	90.48
**390**	3.76	26.99	22.98	44.85	121.67	28.16	45.58
**420**	3.25	39.54	20.74	44.47	110.68	15.99	40.83

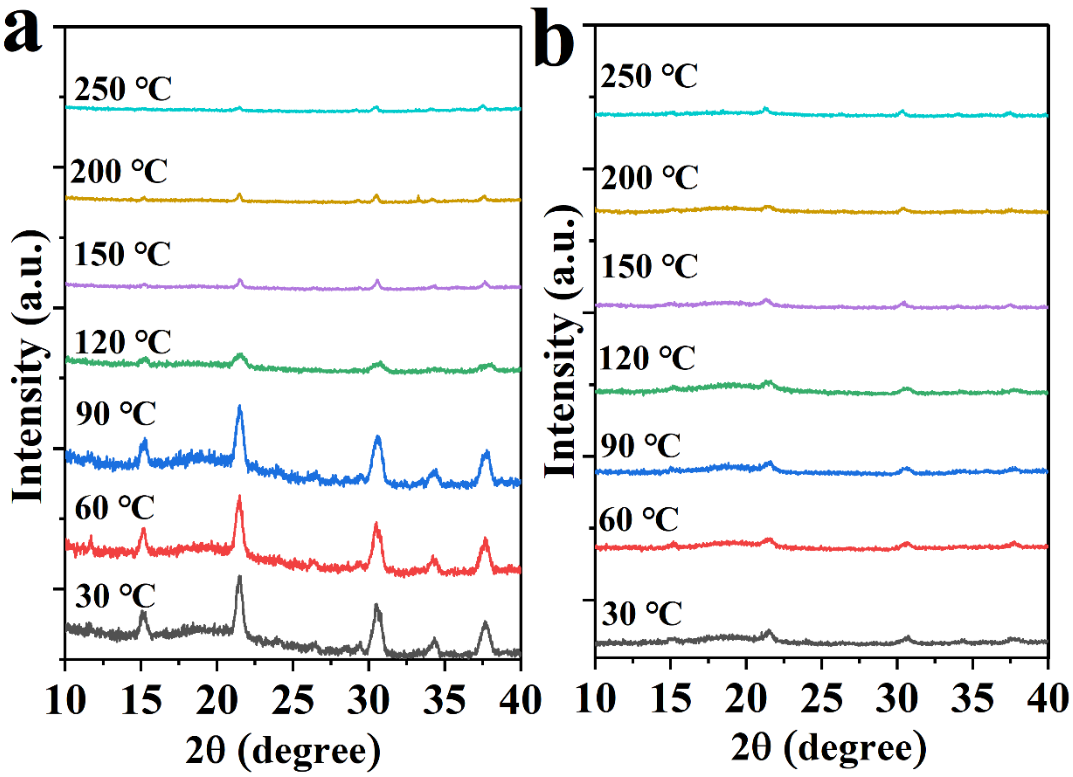

Supplementary Fig. 31. Temperature-dependent XRD patterns. (a) $CsPbBr_3$ fibers and (b) $CsPbBr_3$@HPβCD@PFOS fibers.

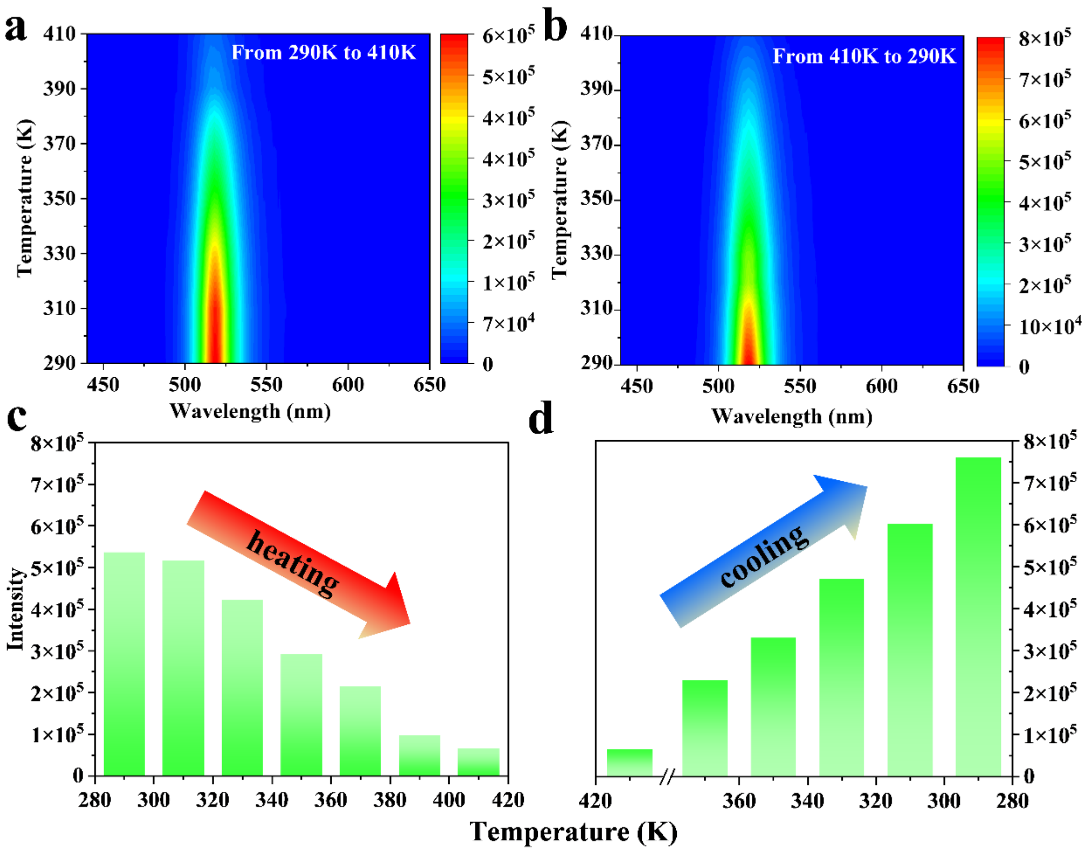

Supplementary Fig. 32. The temperature-dependent PL intensity mapping and evolution of $CsPbBr_3$@HPβCD@PFOS fibrous film, which was heated from 290 K to 410 K (a, c) and cooled down from 410 K to 290 K (b, d).

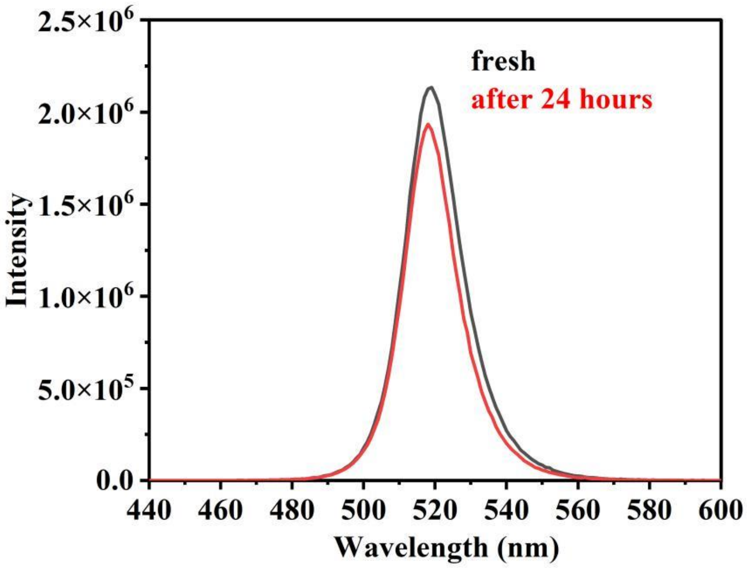

Supplementary Fig. 33. The PL intensity of the $CsPbBr_3$@HPβCD@PFOS textile before and after 24 h UV light irradiation.

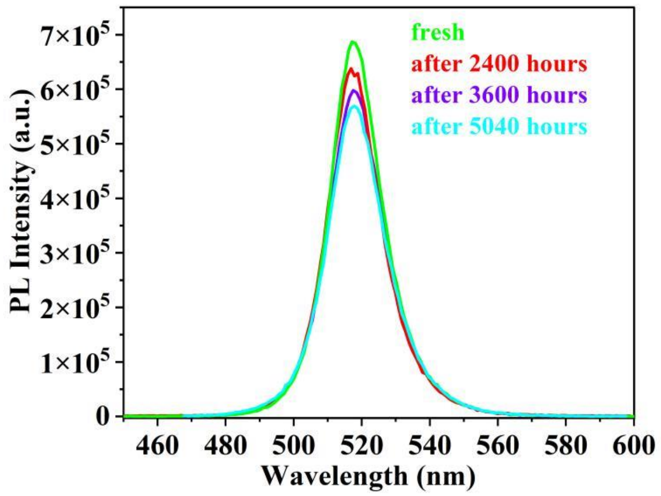

Supplementary Fig. 34. PL intensity evolution of the as-prepared PLT stored in ambient air for different durations.

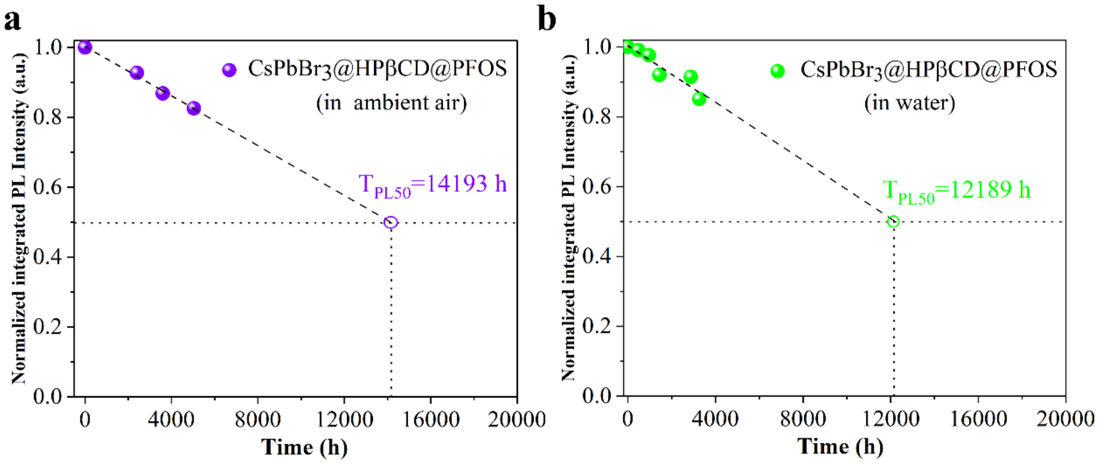

Supplementary Fig. 35. T_{PL50} evaluation of the as-prepared PLTs under ambient storage (a) or water immersion (b).

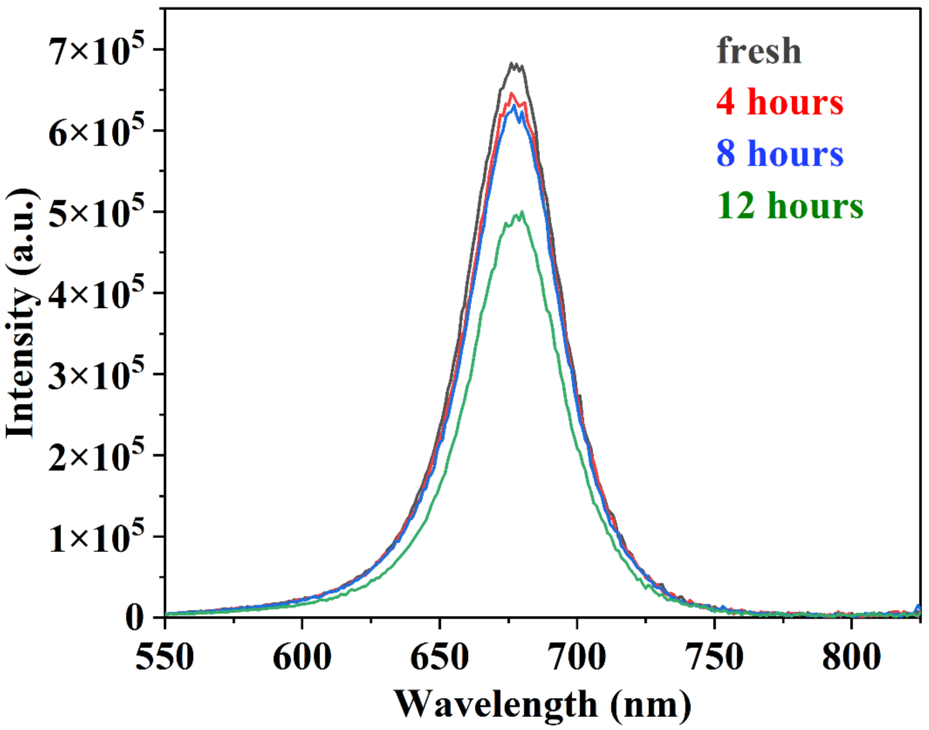

Supplementary Fig. 36. The PL intensity evolution of the red-emitting $CsPb(Br/I)_3$@HPβCD@PFOS textile after UV light irradiation for 12 hours.

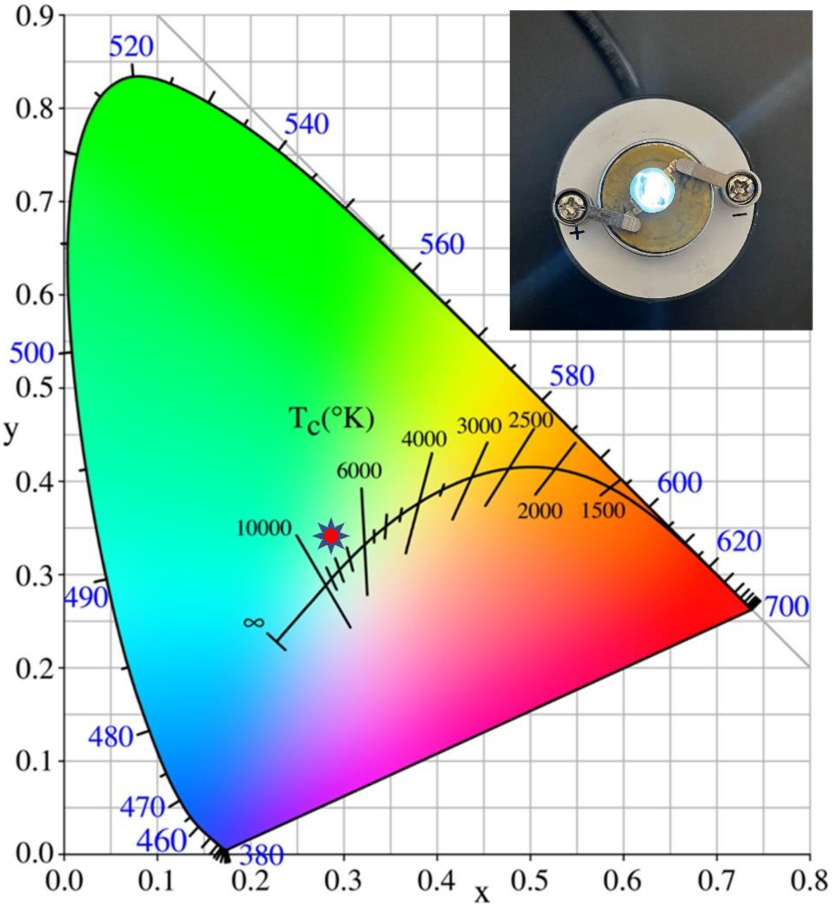

Supplementary Fig. 37. The 1931 CIE chromaticity coordinates of white LED fabricated with three kinds of different-colored PLTs. (The working voltage is 5 V and the driving current is 100 mA.)

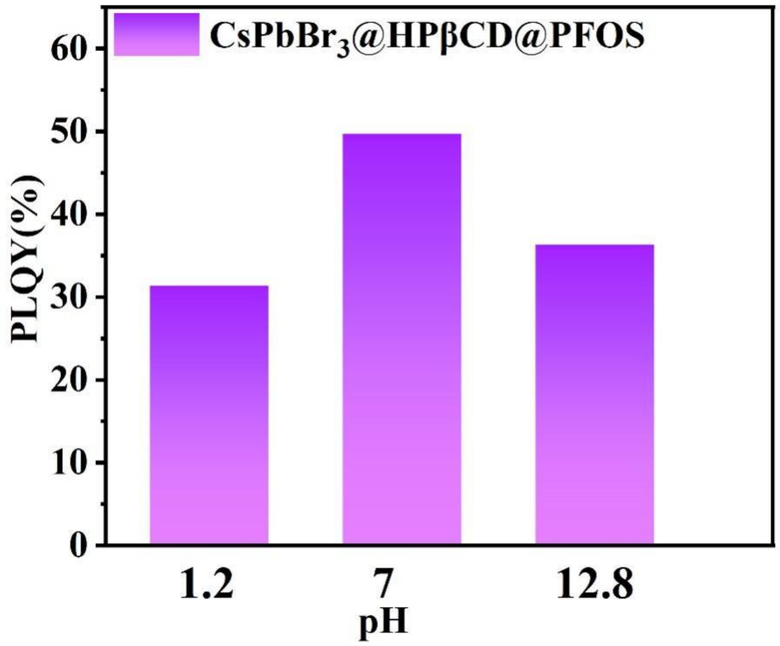

Supplementary Fig. 38. The PLQYs of PLTs in acidic, neutral and basic water solution.

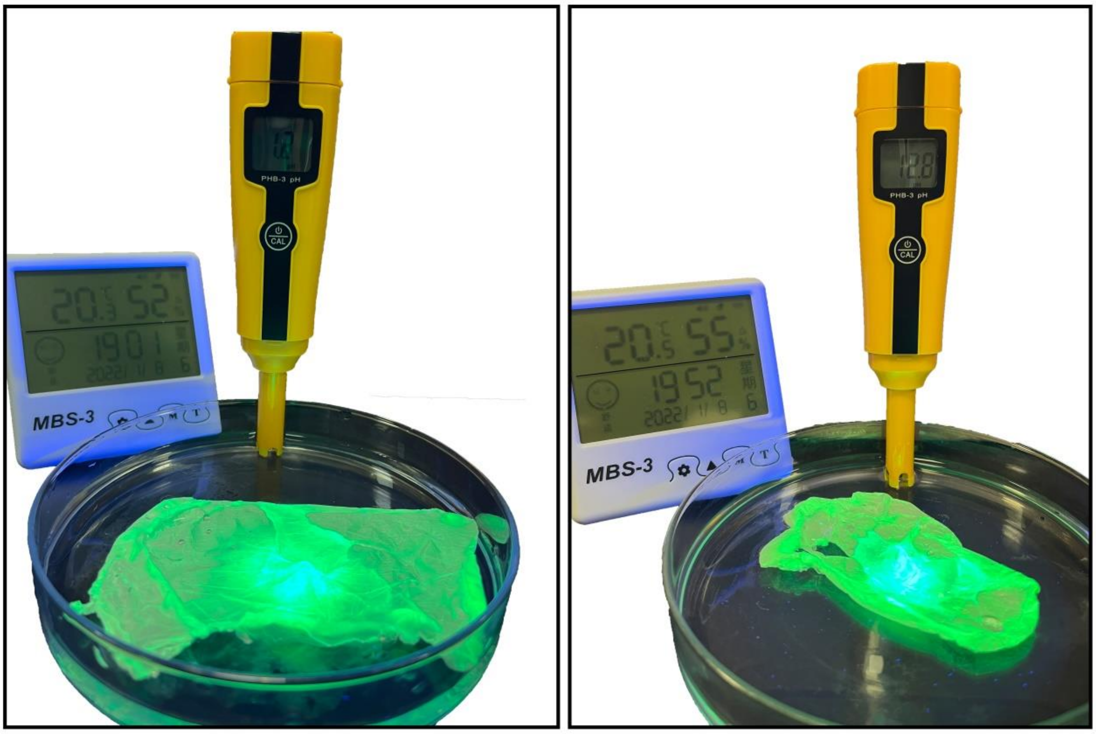

Supplementary Fig. 39. Images of PLTs under UV light irradiation immersed in acidic (pH = 1.2) (left) or basic (pH =12.8) water (right).

Supplementary Table 10. A summary of the state-of-the-art luminescent perovskite nanocrystals-based composites and corresponding large-area luminescent films.

Samples	Methods	Stability			PLQY (%)	PL FWHM (nm)	Pb^{2+} ions leakage	Film area	Ref.
		Water/ polar solvent	High Temperature	UV irradiation					
PS@CsPbBr$_3$@cyclodextrin@PFOS	Single-nozzle electrospinning	Preserved > 85% of PL intensity after 3260 h water immersion	stable up to 250 ºC	Preserved 90.3% of PL intensity under 120 mW cm^{-2} irradiation for 24 h	49.7 (film)	16.96	< 3.94 ppt after dynamic water scouring for 3,300 h	Up to 42000 cm^2 of flexible luminescent textiles	**This work**
$(CsPbBr_3)_{0.25}(a_gZIF\text{-}62)_{0.75}$	Liquid-phase sintering	Preserved 80% of PL intensity after 10000 h water immersion	Preserved over 80 % PL intensity after 100 ºC in Ar or 80 ºC in air	Preserved 90% of PL intensity under 57 mW cm^{-2} irradiation for 5000 s	46.9 (solid)	24.4	Below the detection limit of ICP-OES after 10,000 h immersion in water	N/A	*ref.*14
$CsPb(Br_{1-x}I_x)_3$ in glass	Direct lithography	Exhibit notable stability against organic solution	stable up to 250 ºC	No change in PL intensity under 2 W cm^{-2} irradiation for 12 h	Not reported	N/A	N/A	N/A	*ref.*15
$CsPbBr_3$/ Cs_4PbBr_6	Evaporation of nonstoichiometric CsBr and $PbBr_2$	N/A	N/A	N/A	40.8 (film)	N/A	N/A	90 cm^2	*ref.*20
$CsPbBr_3$@ SiO_2/ZrO_2/ PMMA	sol-gel reaction	N/A	Emission band changes upon increasing the temperature from RT to 120 ºC	N/A	65 (powder)	16	N/A	N/A	*ref.*21
$CsPb(Br_{0.84}Cl_{0.16})_3$	Blade coating	N/A	N/A	N/A	16.18 (film)	N/A	N/A	54 cm^2	*ref.*22
$MAPbBr_3$ @Pb-MOF	Rotary evaporation, followed by washing with diethyl ether, and then dried under vacuum	N/A	N/A	N/A	39.6 (powder)	25	N/A	N/A	*ref.*23
$CsPbBr_3$/ Cs_4PbBr_6	heterogeneous liquid/solid phase interfacial thermal reaction	N/A	stable up to 200 ºC	The PL intensity decreased to 29.4% under 24 mW cm^{-2} irradiation for 12 h.	94 (powder)	20	N/A	N/A	*ref.*24

$CsPbBr_3$ @TFE-HE fluorinated ester	Sonication, UV photo-polymerization	Emiting bright green light after being immersed in water for a month	N/A	N/A	49.92 (film)	19	N/A	N/A	*ref.*33
$CsPbBr_3$@PMMA@TPU	Electro-microfluidic spinning	Preserved 82% of PL intensity after 120 h water immersion	N/A	N/A	39 (film)	23	N/A	N/A	*ref.*35
$MAPbX_3$, (X=Cl, Br, and I)@PAN	Fiber-spinning chemistry based on a microfluidic blow spinning	Preserved 92% of PL intensity in water for 7 days	The PL intensity decreases by 20 % in the first 0.5 h at 150 °C	N/A	71 (film)	21	N/A	120 ×30 cm	*ref.*37
PAN/$MAPbBr_3$ nanofiber films	Fiber spinning chemistry	Preserved 35% of PL intensity in water for 120 h	The PL intensity declined by 25% in the first 1 h at 120 °C	N/A	58 (film)	23	N/A	N/A	*ref.*38
$CsPbX_3$@PS	Swelling-shrinking strategy	Preserved 20-30% of PL intensity in water for 30 days.	$CsPbBr_3$@PS composites are stable under temperature range from 100 to 190 °C	Preserved 40% of PL intensity under UV-light irradiation for 15 days	68 (powder)	N/A	Leaking Pb^{2+} concentration is 1.931 ppm after stirring for 24 hours.	N/A	*ref.*39
$MAPbBr_3$ @polymer gels	Fast one-pot photopolymerization method	Preserved 95.3% of PL in water for 16 days	PL intensity was well maintained at 100 °C, but dropped significantly at 150 °C in 1 hour	N/A	N/A	17-22	Leaking Pb^{2+} concentration is 0.677 ppm after 1 day and 0.749 ppm after 20 days	20 mm × 20 mm	*ref.*40
$CsPbX_3$@polymer fibers	Electrospinning	Retained over 70% of PLQY after being immersed in water for 192 h	PL QY maintained over 50% after being heated at 80 °C for 120 min	N/A	48 (film)	24	N/A	10 cm × 20 cm	*ref.*41
$MAPbBr_3$ quantum wires in porous alumina membranes	Close-spaced vapour reaction	Preserved 99% of PL intensity after 5 days in ambient air with ~45-55% relative humidity	N/A	N/A	92 (arrays)	19	N/A	4 inch wafer (~81 cm^2) and ~7 cm^2 flexible LED device	*ref.*52
