# Supplementary material for: Large-area waterproof and durable perovskite luminescent textiles
Source: Nat Commun. 2023 Jan 16;14:234. doi: 10.1038/s41467-023-35830-8 (PMC9842651; doi:10.1038/s41467-023-35830-8)
Supplement: Supplementary file 2 — Description of Additional Supplementary Files [file 41467_2023_35830_MOESM2_ESM.pdf]

Description of Additional Supplementary Files

File Name: Supplementary Movie 1
Description: Electrospinning PLTs process.

File Name: Supplementary Movie 2
Description: The luminescent properties of folded $CsPbBr_3$@HPβCD@PFOS-based PLT.

File Name: Supplementary Movie 3
Description: The PLT under dynamic seawater scouring and its continuous luminescence.
